# Unraveling the corrosion-mitigating action of cyclobenzaprine hydrochloride on carbon steel in acidic media: integrated experimental and multiscale molecular perspectives

**DOI:** 10.1038/s41598-026-52414-w

**Published:** 2026-06-05

**Authors:** Magdy A. M. Ibrahim, Manal A. El Sayed, Ibrahim H. Elshamy, Shimaa Abdel Halim

**Affiliations:** 1 Chemistry Department, Faculty of Science, Ain Shams University, Abbassia, Cairo 11566 Egypt; 2 Chemistry Department, College of Science, Qassim University, 51452 Buraidah, Saudi Arabia; 3 Chemistry Department, Faculty of Education, Ain Shams University, Roxy, Cairo 11711 Egypt

**Keywords:** Cyclobenzaprine, Corrosion inhibitor, Electrochemical impedance spectroscopy, SEM/EDS, Polarization, Electrode, Chemistry, Materials science

## Abstract

Using a combination of computational and experimental methods, the corrosion protection effectiveness provided by cyclobenzaprine (CBA) for carbon steel (CS) in a 1.0 M hydrochloric acid solution was systematically examined. The corrosion protection efficiency and electrochemical behavior of CS with and without different concentrations of CBA were assessed using gravimetric measurements, polarization studies, impedance measurements, and the evaluation of the open circuit potential. The addition of CBA resulted in a notable decrease in double-layer capacitance (C_dl_) and an increase in charge transfer resistance (R_ct_), a considerable increase in the relaxation time constant (τ), as well as a considerable decrease in corrosion rate and corrosion current density, according to gravimetric and electrochemical data. The formation of a surface-bound shielding layer on the CS was demonstrated by surface characterization using SEM/EDS, AFM analysis, wettability assessment via contact angle evaluation, and UV–visible spectroscopic examination. At 192 μmol L^−1^, the most effective corrosion protection of ~ 96% was achieved. The results showed that CBA functioned as an effective mixed-type inhibitor. Predominant physical adsorption was indicated by the corrosion inhibition efficiency, which improved with higher inhibitor dosage but decreased as the temperature rose. The thermodynamic and kinetic analyses showed spontaneous adsorption, following the Langmuir isotherm. The electronic characteristics and active adsorption centers of CBA were elucidated through density functional theory calculations, Fukui function examination, and natural bond orbital investigation. Additionally, the significance of π–electron systems and heteroatoms in surface interactions was emphasized.

## Introduction

Carbon steel (CS) is widely used in chemical and petrochemical industrial applications owing to its low cost, good mechanical properties, and ease of fabrication^1–4^. It is commonly employed in pipelines, heat exchangers, boilers, reactors, and storage systems. However, when exposed to aggressive environments, particularly acidic media, CS exhibits a high tendency to corrosion, which leads to significant economic losses, safety concerns, and environmental risks^5–8^. Hydrochloric acid (HCl) solutions are widely utilized in engineering activities, including pickling with acid, scale removal, and acid treatment of oil wells, where severe corrosion of CS often occurs^9–11^. Consequently, the development of effective corrosion control strategies for CS in acidic environments remains an important industrial and scientific challenge.

The need for more effective and sustainable substitutes is highlighted by the numerous documented corrosion inhibitors’ toxicity, environmental issues, or poor protection in harsh acidic environments. Among the various control strategies for corrosion, the application of organic corrosion inhibitors is regarded as one of the simplest and most economical approaches. These inhibitors work by adhering to the metallic surface and creating a barrier that prevents cathodic H_2_ evolution and/or anodic dissolution of the metal^12–15^. Numerous studies have reported that heterocyclic organic molecules containing nitrogen, sulfur, and oxygen atoms, along with π–electron-rich aromatic frameworks, can provide high corrosion resistance. Examples include pyrazole, triazole, benzimidazole, pyridine, and their derivatives^16–20^. Despite their effectiveness, many conventional inhibitors suffer from drawbacks such as toxicity, limited biodegradability, and poor solubility under corrosive conditions, which restrict their large-scale industrial application.

Pharmaceutical compounds are potentially suitable for high-performance corrosion protection because they have superior molecular stability, solubility, and specified adsorption sites compared to traditional inhibitors. Recently, considerable attention has been directed toward the advancement of eco-friendly corrosion inhibitors. Pharmaceutical compounds have attracted considerable attention as viable alternatives, owing to their low toxicity and high solubility, and well-defined molecular structures^21,22^. Additionally, the reuse of expired or unused drugs as corrosion inhibitors provides an environmentally sustainable approach to managing pharmaceutical waste. The effectiveness of these compounds is largely due to adsorption at active sites, including heteroatoms with lone pair electrons and conjugated π–electron systems, which facilitate strong interactions with the metal surface^23^. Organic inhibitors interact with the metal surface through physical adsorption and chemical adsorption, or a combination of the two processes, and their adsorption behavior can be described using isotherm models such as Langmuir, Temkin, and Frumkin^24^.

Few investigations combine experimental electrochemistry with quantum chemical calculations and molecular dynamics simulations, despite an increase in publications on drug-based inhibitors. Cyclobenzaprine hydrochloride (CBA) is a therapeutic agent that functions as a muscle relaxant while providing analgesic and anti-inflammatory effects. Structurally, CBA possesses an extended conjugated system, multiple aromatic rings, and a tertiary amine nitrogen, all of which are favorable for corrosion inhibition. The nonbonding electrons on the N atom enable coordination with vacant d-orbitals of iron, while π–metal interactions arising from aromatic rings further enhance adsorption strength. Moreover, the bulky tricyclic structure of CBA is expected to promote the advancement of a structurally compact protective film layer that efficiently isolates active corrosion regions. Despite these advantageous characteristics, the utilization of CBA as an acidic corrosion inhibitor for CS remains unreported.

To the authors’ knowledge, this is the first systematic study of CBA as a corrosion inhibitor for CS in strongly acidic media. Accordingly, the present study aims to provide a comprehensive experimental and predictive evaluation or modeling evaluation of CBA as a corrosion-mitigating agent for CS in 1.0 M HCl. The inhibitor effectiveness and adsorption behavior of CBA are examined through gravimetric analysis in conjunction with electrochemical methods, and surface characterization methods, augmented by sophisticated computational methodologies such as density functional theory (DFT) and natural bond orbital (NBO) analysis. This integrated approach gives a detailed understanding of the corrosion inhibition behavior of CBA, highlighting its ability to serve as a strong, environmentally compatible inhibitor in acidic conditions.

## Experimental

### Materials

The impact of the inhibitor (CBA) on CS in acidic environments was analyzed using mass loss measurements, comparing specimens without inhibitor to those treated with various concentrations. Specimens with dimensions of 1.4 × 1.9 × 0.1 cm (exposed surface area: 0.266 cm^2^) were used for mass-loss experiments. In this study, commercially available steel samples were utilized. Energy-dispersive X-ray spectroscopy (EDS) was used to describe the samples’ chemical composition. The chemical analysis of the CS was composed of (wt.%): 1.2 C, 0.7 Al, 0.2 Si, 1.5 Mn, and 96.4 Fe. Steel coupons were abraded sequentially using various grit sizes of SiC paper, followed by polishing with 2000-grit paper. The samples were cleaned with deionized water, then with acetone, and dried under a warm-air stream.

Epoxy cold resin was employed for the preparation of the CS electrodes, and 1.0 cm^2^ of the electrode surface was subjected to the aggressive solution. In electrochemical experiments, CS electrodes were employed as working electrodes. The freshly prepared acid solution was prepared from a laboratory-grade container with a 37% HCl solution and diluted with deionized water. The counter electrode was a Pt wire. All potentials reported in this work are referenced to an Ag/AgCl (saturated KCl) electrode.

CBA was obtained from an Egyptian pharmacy. A Gamry Instrument Potentiostat/Galvanostat/ZRA, model number 1000, was used for the electrochemical tests. The electrochemical parameters were calculated using the Echem Analyst software from Gamry Instruments. The PDPs were conducted at a scan rate of 2 mV s^−1^ and a potential window of − 0.7 to − 0.2 V. To minimize prolonged surface modification during polarization in the aggressive acidic medium and to ensure quasi-steady-state conditions, a scan rate of 2 mV s^−1^ was used.

**Fig. 1:**
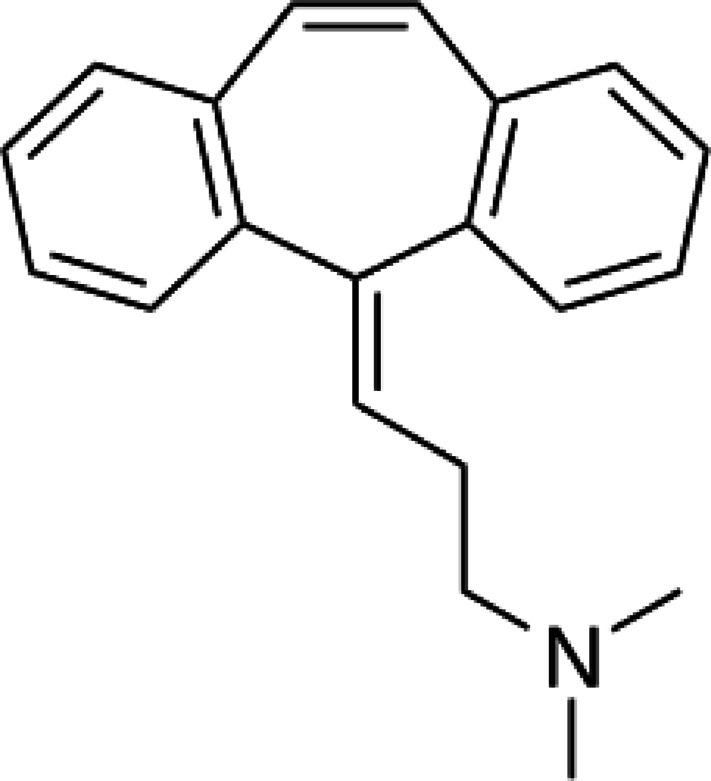
The chemical structure of the cyclobenzaprine (CBA) drug.

The EIS diagrams were plotted with a 5 mV peak-to-peak amplitude and a frequency range of 100,000 Hz to 10.0 mHz after a 900-s exposure to the acid solution. EIS measurements were purposefully performed after 900 s of immersion, even though full OCP stabilization was usually reached after about 1200–1400 s. This exposure time was chosen to probe the electrochemical response at an early yet stable stage of inhibitor film formation; a practice commonly adopted in corrosion studies to capture interfacial processes without compromising data reliability^25^. To guarantee reproducibility, the experiments were carried out three times. The test samples’ surface morphology was analyzed using the JEOL JEM-1200EX II Electron Microscope, a form of scanning electron microscopy (SEM). Figure 1 illustrates the molecular structure of Cyclobenzaprine hydrochloride, which has the chemical formula C20H21N.HCl and a molecular weight (MW) of 311.9. Cyclobenzaprine hydrochloride (CBA. HCl) is a white, crystalline tricyclic amine salt. To be used as a corrosion inhibitor, CBA was prepared in deionized water at concentrations of 32, 64, 96, and 192 μ mol L^−1^. Cyclobenzaprine hydrochloride was fully soluble at all concentrations studied at ambient temperature.

### Quantum chemical studies

#### Theoretical computations

Density functional theory (DFT) simulations were conducted to examine the corrosion inhibition properties of CBA at the molecular scale. To verify the stability of the optimized structure, GaussView 5.0.9 was used for geometry optimization and vibrational frequency analyses. The B3LYP hybrid exchange–correlation functional and the 6–311++ G(d, p) basis set, as implemented in the Gaussian 09 program package, were used in all computations^26^. The energies of the highest occupied and lowest unoccupied molecular orbitals (E_HOMO_ and E_LUMO_), energy gap (ΔE), dipole moment (μ), ionization potential (I), electron affinity (χ), electronegativity (φ), chemical hardness (ψ), softness (S), electrophilicity index (ω), nucleophilicity index (ε), electro-donating (ω^−^) and electro-accepting (ω^+^) powers, back-donation energy (ΔE), fraction of electron transfer (ΔN), and inhibitor–metal interaction energy (ΔE_*Fe/CBA*_). The adsorption tendency and inhibitory effectiveness of the inhibitor were evaluated using these criteria. Fukui function analysis^27–29^ was used to identify local reactive areas. Furthermore, charge distribution and donor–acceptor interactions pertinent to the inhibitor-metal system were investigated using Natural Bond Orbital (*NBO*) analysis.

#### NBO analysis

To clarify the electronic properties controlling the adsorption and inhibition behavior of CBA, natural bond orbital (NBO) analysis was carried out. Non-covalent effects were ascribed to interactions involving vacant antibonding or Rydberg orbitals, whereas covalent interactions were explained in terms of occupied NBOs connected to the natural Lewis structure^30,31^. To quantify charge-transfer interactions pertinent to inhibitor–metal bonding, donor–acceptor interactions between filled (Lewis-type) and empty (non-Lewis) orbitals were examined. The related stabilization energy, *E*^(2)^, was computed using second-order perturbation theory (Eq. 1).

1 $$E^{\left( 2 \right)} = \Delta Ei{\mathrm{j}} = qi\left( {F\left( {i{\mathrm{j}}} \right)2/\varepsilon j - \varepsilon i} \right)$$

## Results and discussions

### Weight loss (WL) measurements

**Fig. 2:**
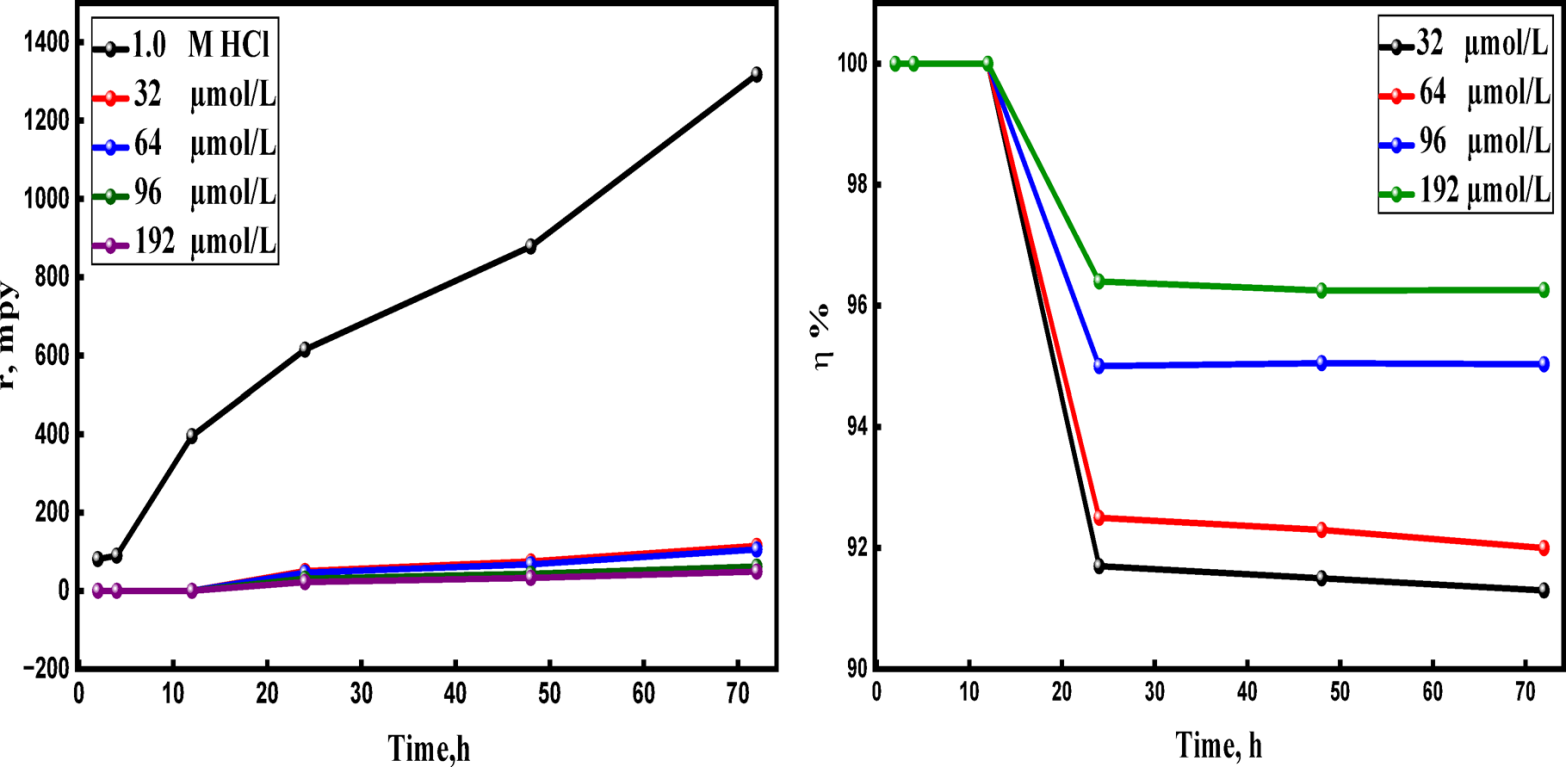
The variation of r of CS and η values of the studied CBA with time.

The corrosion rate (r), inhibition efficiency (η%), and surface covering fraction (θ) by CBA molecules were computed using the following formulas^32^:

2 $$r \left( {mpy} \right) = \frac{{534 \times {\mathrm{W}}}}{{\uprho \times {\mathrm{A}} \times {\mathrm{t}}}}$$

3 $${\uptheta } = \frac{{{\text{Wo }}\;\;{\mathrm{W}}}}{{{\mathrm{Wo}}}}$$

4 $$\upeta \% =\uptheta \times {1}00$$

where A is the exposed CS surface area (in^2^), r is the corrosion rate (rpm), t is the immersion period (hours), W weight loss (mg), and 534 is the unit conversion constant for mpy. Using the weight loss data, the surface coverage θ can be calculated (Eq. 3). Where w and w_o_ stand for the WL values with and without inhibitor, respectively^33^. As seen in Fig. 2, gravimetric analysis was performed in a 1.0 M HCl solution to assess r values below various contact times and concentrations of the examined CBA at 298 K.

Table 1 displays the corrosion rate (r) and inhibition efficiency (η%) of CS in 1.0 M HCl with and without different CBA concentrations at various immersion periods. Because of the aggressive nature of the acidic medium and ongoing metal dissolution, the corrosion rate in the uninhibited solution significantly increased with immersion time. On the other hand, at all concentrations and exposure times, the corrosion rate was greatly reduced by the addition of CBA. The corrosion rate remained almost completely inhibited at short immersion times (2–12 h), with an inhibition efficiency of about 100%, indicating quick adsorption of CBA molecules and efficient surface coverage^34,35^. Longer immersion times (24–72 h) resulted in a slight increase in corrosion rate, but high inhibition efficiencies were maintained, especially at higher CBA concentrations, where η% remained above 95%. This behavior suggests good stability of the adsorbed inhibitor film, with improved protection at higher concentrations due to enhanced surface coverage and stronger adsorption. Overall, the results demonstrate that CBA provides efficient and durable corrosion protection for CS in acidic media.

**Table 1:** WL measurements of CS in 1.0 M HCl solution and after the addition of CBA inhibitor.

[CBA] (µmol L^−1^)	2 h	η%	4 h	η%	12 h	η%	24 h	η%	48 h	η%	72 h	η%
	r (mpy)		r (mpy)		r (mpy)		r (mpy)		r (mpy)		r (mpy)	
0.0	80.9	–	89.6	–	395.2	–	615	–	878.38	–	1317.5	–
32	0	100	0	100	0	100	50.5	91.7	74.81	91.5	114.64	91.3
64	0	100	0	100	0	100	46.3	92.5	67.63	92.3	105.4	92.0
96	0	100	0	100	0	100	30.75	95.0	43.47	95.1	61.9	95.0
192	0	100	0	100	0	100	21.95	96.4	33	96.3	49.07	96.3

### Measurements of potentiodynamic polarization (*PDP*)

**Fig. 3:**
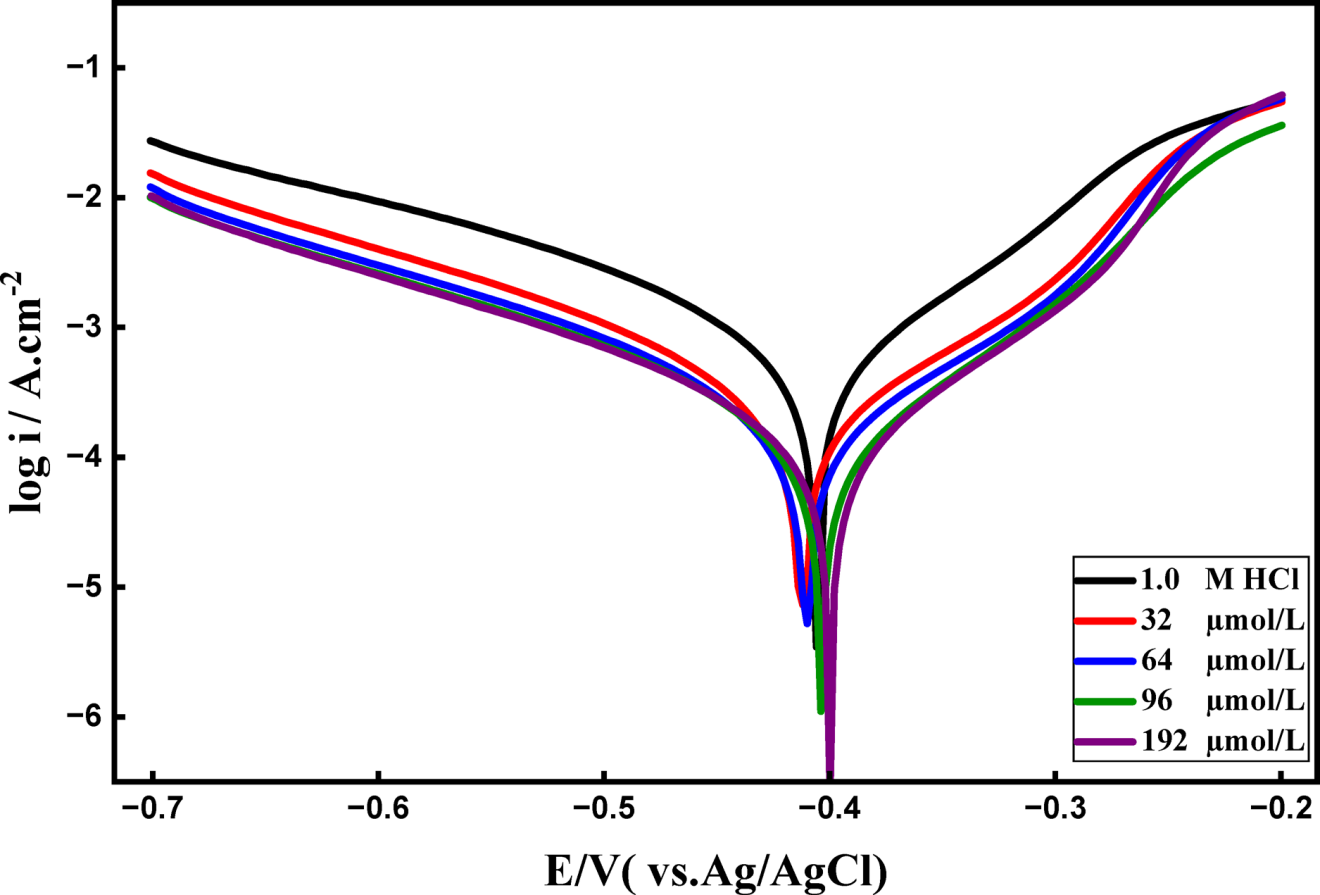
Potentiodynamic polarization curves of CS electrodes at 298 K with a scan rate of 2 mV s^−1^ in 1.0 M HCl solution and with various CBA concentrations.

The polarization technique was employed to investigate how CBA affected the resistance to corrosion of the CS. Based on modifications, inhibitors are typically categorized as cathodic, anodic, or mixed within the polarization curves following the inhibitor behaviour^36,37^. Figure 3 shows the Tafel polarization curves for CS after immersion in HCl at 1.0 M with different CBA concentrations at 298 K. The cathodic and anodic Tafel slopes (βc and βa), corrosion potential (E_corr_), and corrosion current (i_corr_) were calculated using Tafel plots. Equation (5) was then used to get the inhibition efficiency percentage. Table 2 is a tabulation of the outcomes. Table 2 and Fig. 3 make it clear that the addition of CBA at every concentration examined resulted in a notable decrease in both the rate of corrosion and the corrosion current (i_corr_). Additionally, as the inhibitor’s concentration rose, so did its percentage efficiency. The corrosion potential (E_corr_) value changed significantly compared to the blank. The simultaneous and nearly equal influence on both anodic and cathodic polarizations shows how CBA affects metal dissolution, hydrogen development, and both anodic and cathodic processes. Variations in βc are associated with the inhibition of the cathodic hydrogen evolution reaction by surface blocking. In contrast, changes in βa are interpreted in terms of hindered iron dissolution and surface film development. The literature report claims that^38^, when the corrosion potential exceeds ± 85 mV in comparison to the corrosion potential of the blank, the inhibitor can be categorized as either anodic or cathodic. The investigation’s extreme displacement, however, is less than ± 85 mV. Therefore, its mixed-type inhibitory impact is implied by this. The measured i_corr_ values were used to calculate the inhibitory efficiency values η%.

5 $$\upeta \left( \% \right) = {\uptheta } \times 100 = \frac{{{\text{icorr }} - {\text{ icorr}}\left( {{\mathrm{inh}}} \right)}}{{\text{ icorr }}} \times 100$$

where *θ* represents the surface covering and i_corr_ and i_corr_(inh) represent the corrosion current densities with and without inhibitor concentrations.

**Table 2:** Tafel kinetic parameters measured at 298 K for a CS in a 1.0 M HCl solution with various CBA concentrations.

[CBA] (µmol L^−1^)	i_corr_ (A cm^−2^)	− E_Corr_ (/V)	β_a_ (V dec^−1^)	β_c_ (V dec^−1^)	Corr rate (mpy)	$\Theta$	η_PDP_ (%)
0.0	5.42 × 10^−4^	0.406 ± 0.05	0.097 ± 0.03	0.154 ± 0.03	69.75 ± 1.4	–	–
32	2.05 × 10^−4^	0.412 ± 0.05	0.117 ± 0.05	0.113 ± 0.04	26.42 ± 1.6	0.621 ± 0.05	62.17 ± 1.5
64	1.32 × 10^−4^	0.411 ± 0.05	0.097 ± 0.03	0.091 ± 0.06	17.00 ± 1.6	0.756 ± 0.03	75.64 ± 1.4
96	1.01 × 10^−4^	0.403 ± 0.05	0.091 ± 0.06	0.083 ± 0.04	12.99 ± 1.7	0.813 ± 0.04	81.36 ± 1.3
192	3.26 × 10^−5^	0.400 ± 0.04	0.030 ± 0.06	0.029 ± 0.03	4.192 ± 1.5	0.939 ± 0.03	93.98 ± 1.2

### Electrochemical impedance spectroscopy (EIS)

Since electrochemical impedance spectroscopy (EIS) is an effective method that can yield detailed information on the corrosion process and the protective performance of inhibitors without destroying the sample, it has been used to investigate corrosion mechanisms. EIS enables us to evaluate the inhibitors’ adsorption performance on the metallic surface through detecting variations in capacitance and charge transfer resistance across a varied spectrum of frequencies. This aids in precisely replicating an adsorption isotherm^39,40^.

**Fig. 4:**
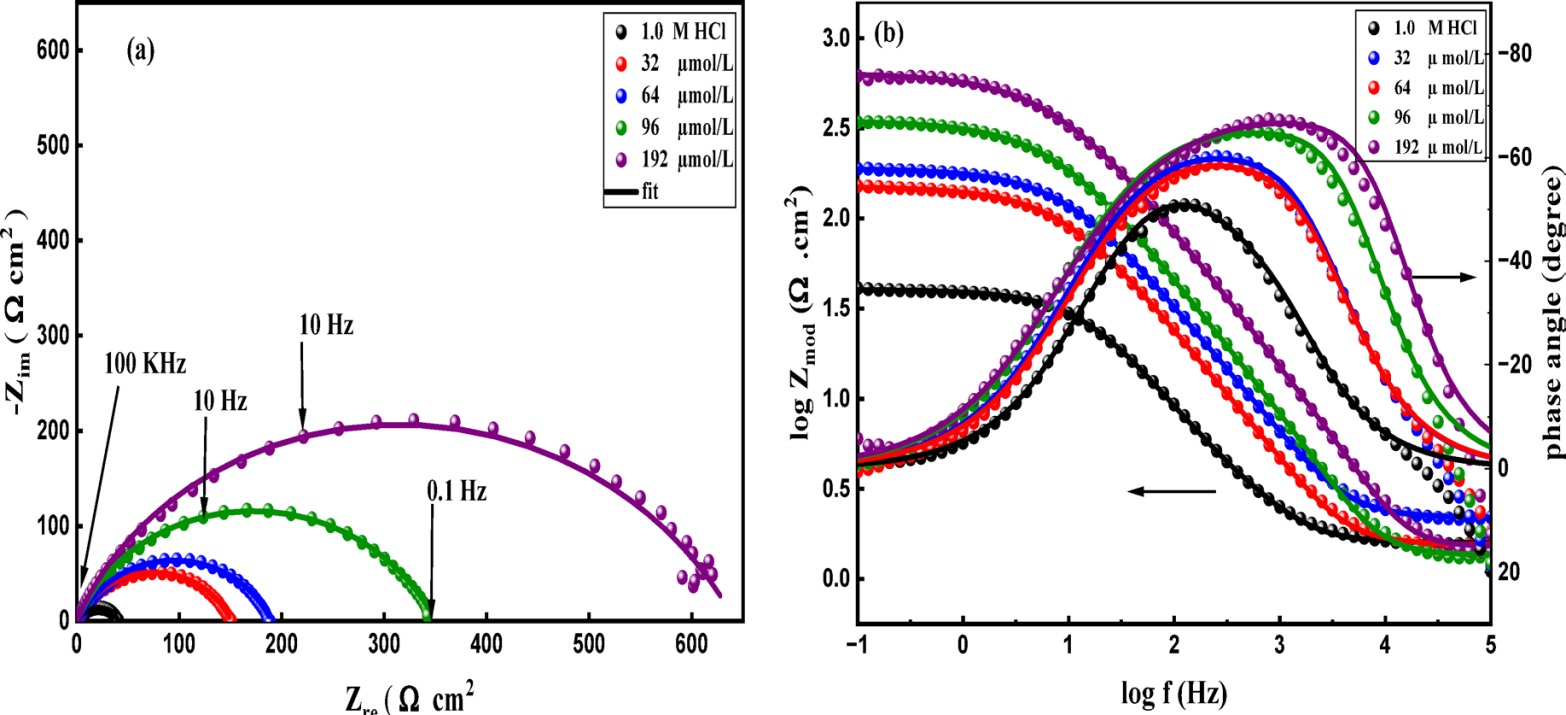
(**a**) Nyquist plots and (**b**) Bode plots of CS data in 1.0 M HCl at various CBA concentrations at 298 K.

Among the numerous components of this circuit are the constant phase element (CPE) associated with the charge-transfer process, the solution resistance (Rs), and the charge-transfer resistance (R_ct_). The double layer at the interface between the charged metal surface and the solution creates an electrical capacitor^41–43^. The following equation yields the CPE:

6 $${\mathrm{Z}}_{{{\mathrm{CPE}}}} = \left[ {{\mathrm{C}}_{{}} \left( {{\mathrm{j}}\omega } \right)^{{\mathrm{n}}} } \right]^{{ - {1}}}$$

where j is the unreal element, and n is the CPE exponent in relation to the surface inhomogeneity. C stands for capacitance, and the angular frequency is denoted by ω. The range of values for n is 0–1. The CPE behaves as a resistor when n = 0 and as an ideal capacitor when n = 1^44^. Figure 4 shows the behaviors of CS immersed in acidic solutions at various CBA concentrations. Surface heterogeneity is revealed by the CPE exponent (n), which represents the extent of departure from ideal capacitive performance. A highly heterogeneous and actively corroding surface is indicated by lower n values in the uninhibited solution. When the inhibitor is added, n rises and gets closer to unity, indicating that a more uniform and compact adsorbed layer has formed. Simultaneously, the stability of the steel/solution interface and the efficient suppression of charge-transfer processes are confirmed by the decrease in C_dl_ and the increase in R_ct_ and relaxation time constant (τ). Surface heterogeneity brought on by insufficient inhibitor coverage is responsible for the departure from optimal capacitive behavior seen for partially inhibited surfaces.

The kinetics of the corrosion process are revealed by the relaxation time constant (τ), which is computed from the fitted EIS parameters (Table 3). The following relationship was used to calculate the relaxation time constant ($\tau$):

7 $$\tau = R_{ct} \times C_{dl}$$

**Table 3:** EIS fitting parameters were derived for a CS in a 1.0 M HCl solution at 298 K with varying CBA concentrations.

[CBA] (µmol L^−1^)	R_s_ (Ω cm^2^)	R_ct_ (Ω cm^2^)	CPE (Ω^−1^ s^n^ cm^−2^)	N	$\tau$ (s)	χ^2^ × 10^−3^	$\Theta$	η_EIS_ (%)	C_dl_ × 10^−5^ (F cm^−2^)
0.0	1.549	38.87	657.0 × 10^−6^	0.792	0.00792	6.020	–	–	25.00
32	1.448	145.6	244.0 × 10^−6^	0.794	0.01456	7.817	0.733	73.30	10.00
64	2.050	183.8	190.6 × 10^−6^	0.785	0.01415	4.460	0.788	78.85	7.70
96	1.199	331.3	121.0 × 10^−6^	0.804	0.01287	11.97	0.882	88.26	5.52
192	1.257	600.7	67.61 × 10^−6^	0.800	0.019823	12.99	0.935	93.52	3.30

The development of a protective adsorbed film causes a slower interfacial charge-transfer process, as evidenced by the considerable increase in τ in the presence of the inhibitor as compared to the uninhibited solution. Non-ideal capacitive behavior and intermediate τ values for partially inhibited surfaces indicate a variety of relaxation durations and heterogeneous surface covering.

**Fig. 5:**
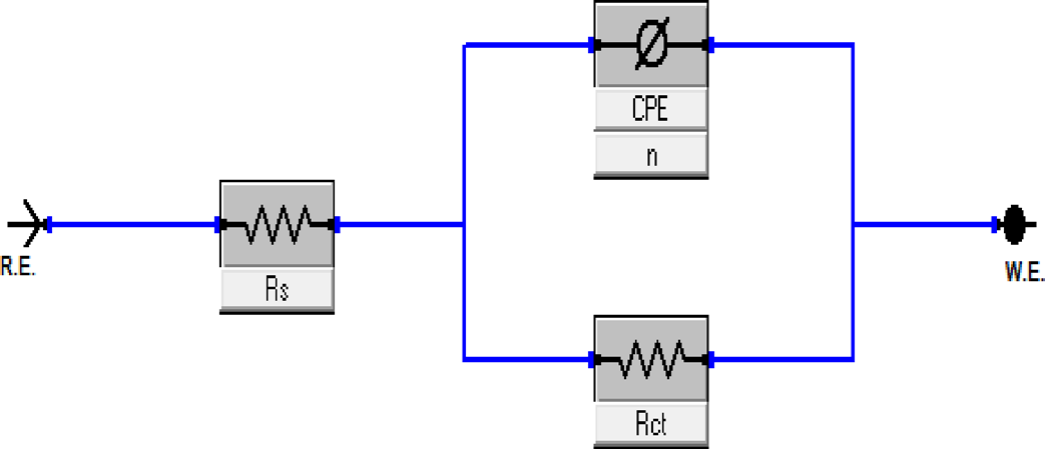
Equivalent circuit model to fit the EIS data obtained at the CS/electrolyte interface.

The Nyquist plot in Fig. 4a, which includes the corresponding diameters, shows that the EIS response curves are semicircular, which does not significantly change as the corrosion inhibitor concentration increases. As a result, charge transfer processes limit the corrosion mechanism in CS and leave it unaltered^45^. The Nyquist graphs show several intricate modifications when the CBA corrosion inhibitor is added. The expanding capacitive loop’s diameter indicates improved impedance. Phase angle changes to a greater negative amount at middle frequencies, indicating improved capacitive behavior. Furthermore, at lower frequencies, the absolute value of impedance increases, suggesting increased corrosion resistance. Because CBA molecules include adsorption centers in their structures, they limit the corrosion of CS, which is probably the cause of these effects. These adsorption sites allow CBA molecules to stick to the surface of steel, creating a layer of protection that stops corrosion^46^. Non-ideal capacitive behavior is seen in Fig. 4, where the loop of capacitors is depicted as a lowered semicircle. A near-linear region with a slope close to − 1 was observed in the Bode magnitude plot, suggesting capacitive control of the electrochemical process. Table 3 displays the phase angles in the middle frequency range. This fluctuation implies an uneven surface of the steel. The reason for the non-uniformity is the frequency dispersion effect, which implies that the surface properties and corrosion response of the steel are not consistent across different frequencies. Different adsorption sites, surface roughness, or additional reasons affecting the impedance readings could cause this non-homogeneity^47^. An equivalent circuit for a one-time constant is generally used to study the concept of consistent corrosion, as shown in Fig. 5. The accuracy of the equivalent circuit fitting for both uninhibited and inhibited systems is illustrated using representative experimental and simulated Nyquist and Bode plots. The validity of the suggested models is confirmed by the strong agreement between simulated curves and experimental data, as well as by low χ^2^ values and modest fitting errors.

On the other hand, the maximum phase angle values were near each other when CBA was added to all the 1.0 M HCl solutions being examined, demonstrating that the stability of the resulting protective films was similar. Larger phase angle values for CBA show that the film that forms in 1.0 M HCl solution provides less protection when the CS is exposed to an aggressive environment. Furthermore, the polarization observations are consistent with the inhibitory efficiency order ascertained by EIS measurements. The following Eq. (8) is used to estimate and report the inhibition efficiency in Table 3^48^:

8 $$\eta { }\left( {{\% }} \right){ } = \frac{{{\mathrm{Rct}} - {\mathrm{R}}^\circ ct{ }}}{{{\mathrm{Rct}}}}\times 100$$

R°_ct_ and R_ct_, respectively, represent charge transfer resistances with and without inhibitors.

As the concentration of CBA inhibitor increases, C_dl_ declines, indicating lesser interfacial charge accumulation due to inhibitor adsorption on the electrode surface and a corresponding decrease in CPE magnitude (Table 3). It is worthwhile noting that all methods consistently show a significant decrease in corrosion rate in the presence of the inhibitor, indicating its good protective effectiveness despite variations in absolute inhibition efficiency.

### OCP measurements

**Fig. 6:**
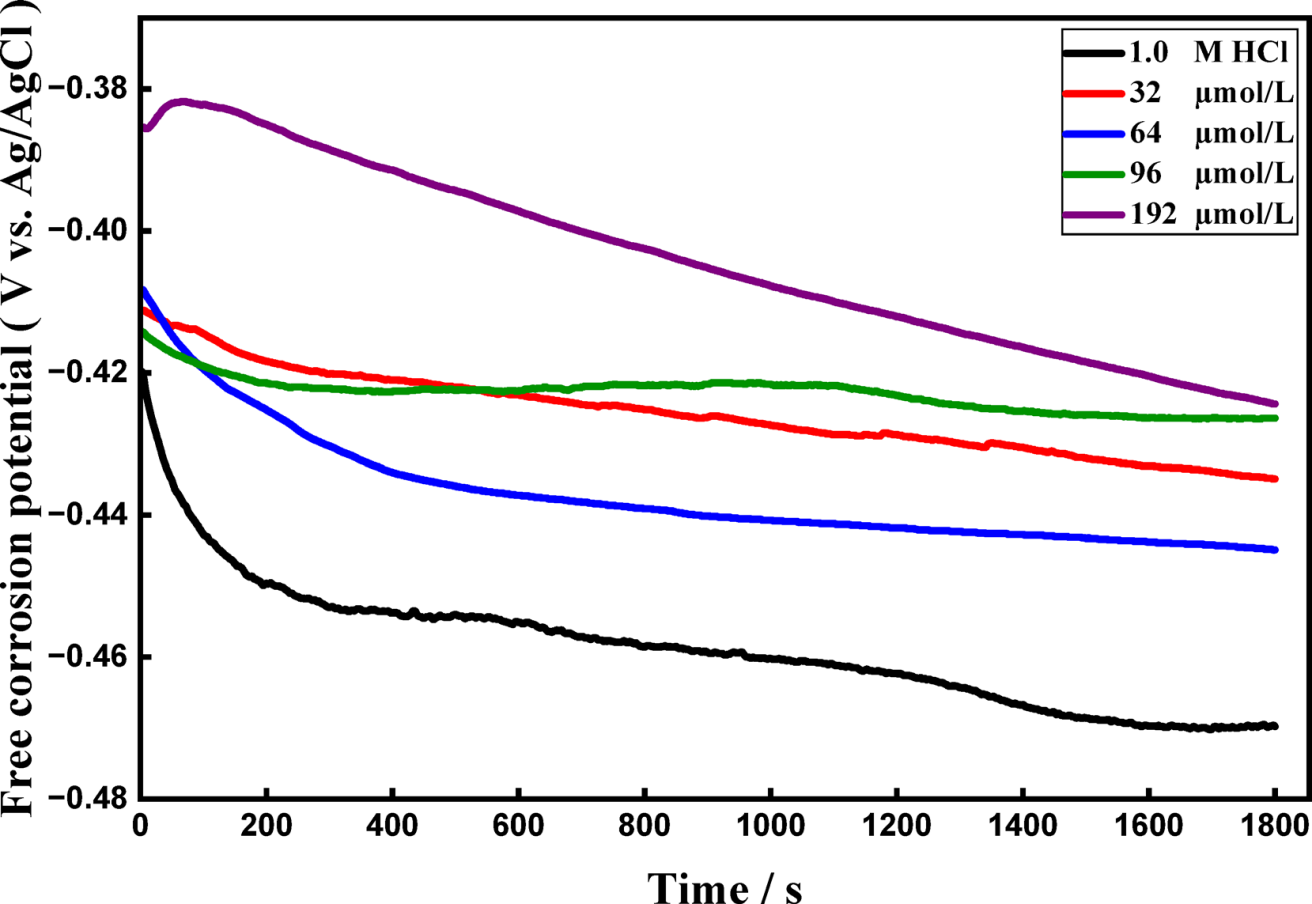
OCP behavior for CS in 1.0 M HCl solution at various CBA concentrations at 298 K as a function of time.

The OCP-time response of CS in a 1.0 M HCl solution at 298 K with and without various CBA concentrations is displayed in Fig. 6. The development of a protective inhibitor film on the steel surface is indicated by the OCP values gradually shifting toward more positive potentials in the presence of CBA after immersion, when compared to the blank solution. This positive shift implies that by inhibiting iron oxidation reactions, CBA has a greater impact on the anodic dissolution of carbon steel^49,50^. The very small potential displacement suggests that rather than acting as a strictly anodic or cathodic inhibitor, CBA acts as a mixed-type inhibitor with a predominant anodic impact. Furthermore, the formation of a steady-state situation at the steel/solution interface is confirmed by the stabilization of the OCP curves after about 1800 s.

### Effect of temperature and kinetics studies

#### Potentiodynamic polarization curves (PDPs) measurements

**Fig. 7:**
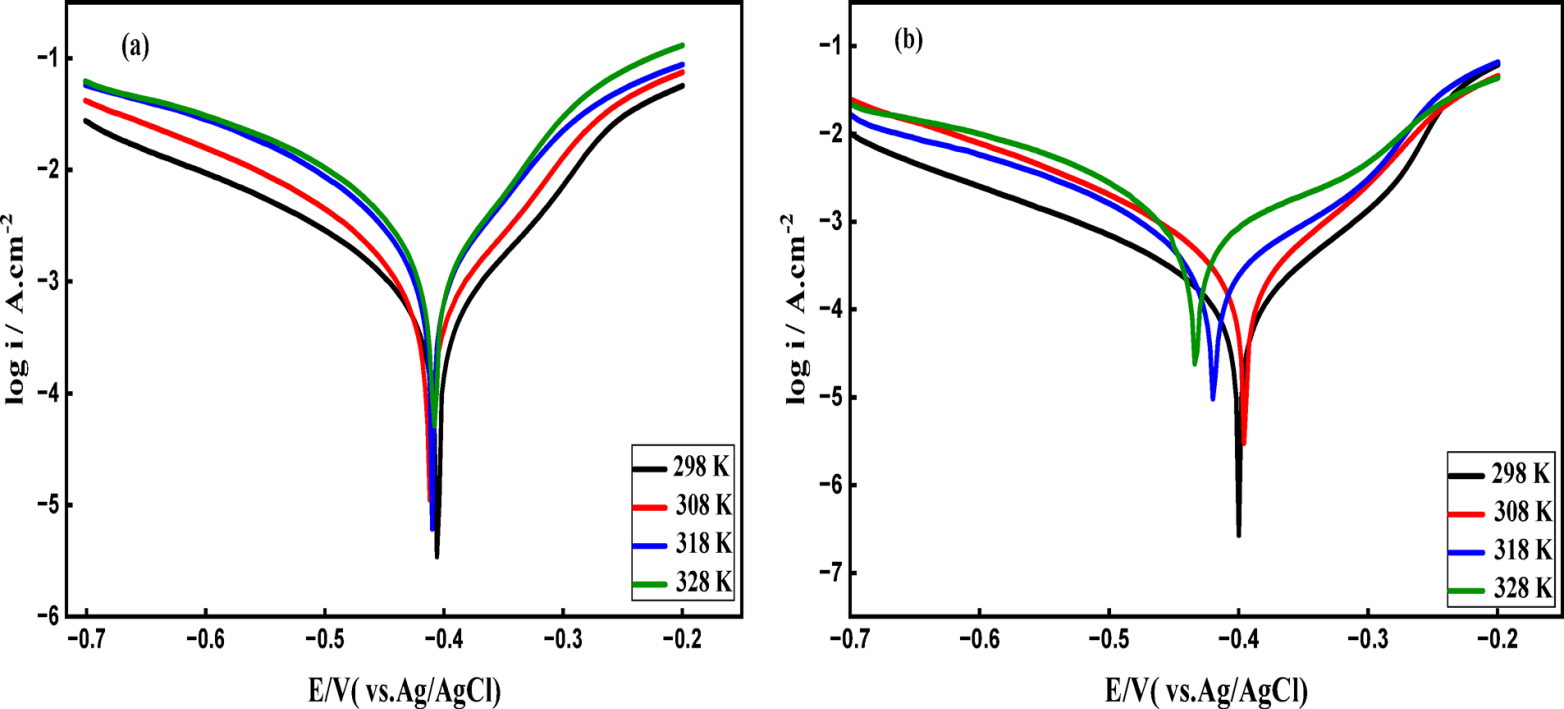
(**a**,**b**) shows the PDPs of CS in a 1.0 M HCl solution at various solution temperatures with and without CBA, obtained at a scan rate of 2 mV s^−1^.

PDPs measurements for the CS under investigation in a 1.0 M HCl, both with and without CBA, were established at the temperature range of 298–328 K in order to offer precise information on the kind of inhibitor adsorption and its efficacy. Regardless of the existence of CBA, the temperature raised the i_corr_ and the r, as observed in Table 4 and Fig. 7a and b. In the absence of CBA, the E_corr_ values varied from − 0.406, 0.412, 0.410 to − 0.408 V as the temperature rose from 298 to 328 K. However, the potential was shifted to higher negative levels (from − 0.400 to − 0.536 V) when the CBA was added to the 1.0 HCl. When the temperature rose, the E_corr_ values moved to increased cathodically. These findings also show that raising the temperature accelerates the anodic and cathodic processes^51–53^. Consequently, corrosion clearly increases with temperature. The βc and βa Tafel lines’ slopes do not change as the temperature rises. This indicates that the CS surface starts to corrode even though the corrosion mechanism is independent of temperature. The findings showed that the inhibitor’s effectiveness declines with temperature. When physically adsorbed, the inhibitor molecules may be connected to the observed behavior since higher temperatures may impede the adsorption procedure. The inhibitor’s adsorption and the creation of a physical block on the metallic surface reduce CBA reactivity in electrochemical operations.

**Table 4:** Tafel kinetic characteristics were measured at various solution temperatures for a CS in a 1.0 M HCl solution with and without the CBA.

Temperature (K)	i_corr_ (A cm^−2^)	− E_Corr_ (/V)	β_a_ (V dec^−1^)	β_c_ (V dec^−1^)	Corr rate (mpy)	$\Theta$	ղ_PDP_ (%)
0.00 µmol L^−1^							
298	5.42 × 10^−4^	0.406	0.097	0.154	69.75	–	–
308	6.98 × 10^−4^	0.412	0.105	0.096	89.94	–	–
318	1.14 × 10^−3^	0.410	0.086	0.080	146.7	–	–
328	1.75 × 10^−3^	0.408	0.099	0.119	225.0	–	–
192 µmol L^−1^							
298	3.26 × 10^−5^	0.400	0.030	0.029	4.192	0.939	93.98
308	1.64 × 10^−4^	0.396	0.072	0.071	21.09	0.765	76.50
318	4.18 × 10^−4^	0.419	0.183	0.121	53.84	0.633	63.33
328	1.27 × 10^−3^	0.536	0.107	0.106	163.1	0.274	27.42

**Fig. 8:**
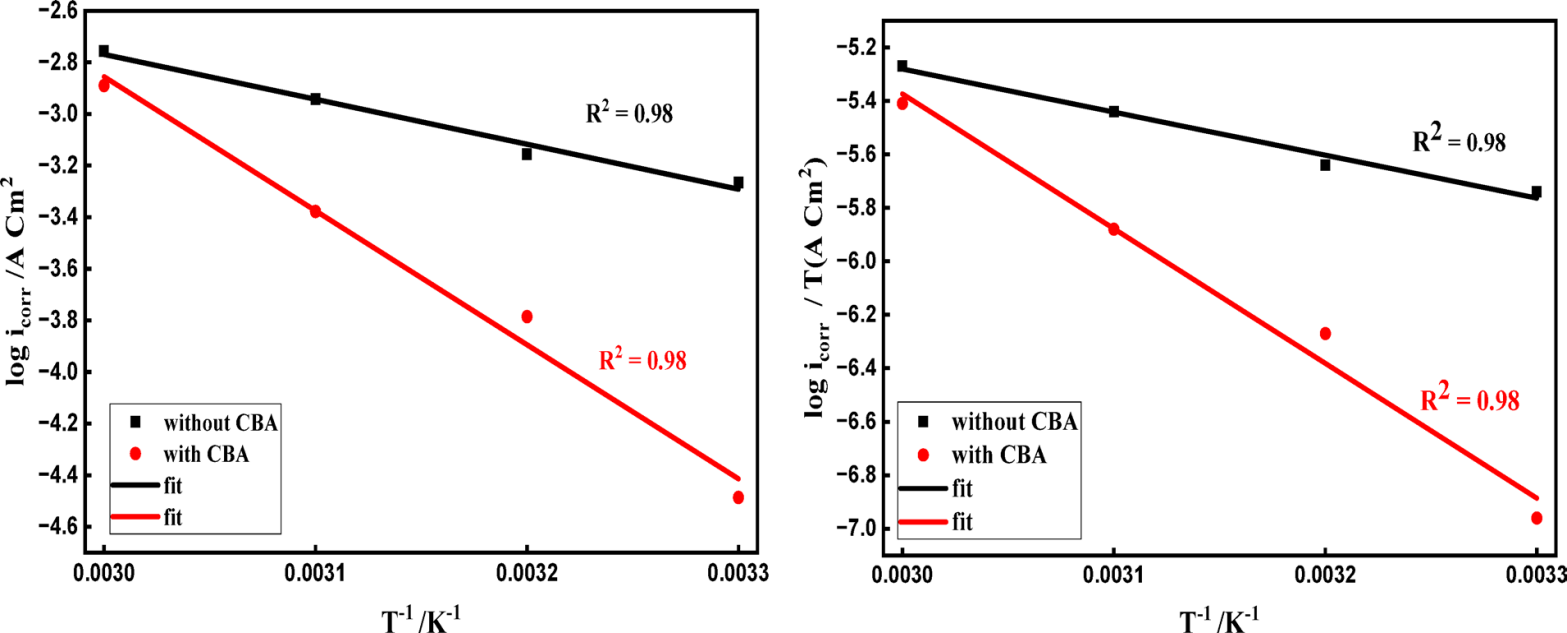
Log i_corr_ vs. T^−1^/K^−1^ Arrhenius plot; log i_corr_/T vs. T^−1^/K^−1^ transition state plot with and without CBA.

This behavior is clearly explained by Eqs. (9) and (10), which stand for the Arrhenius equation and the transition state equation, respectively^54^:

9 $$\mathrm{Log}\, \mathrm{icorr}=\frac{-\mathrm{Ea}}{2.303 \mathrm{R}\mathrm{T}}+\log\mathrm{A}$$

10 $$\mathrm{Log}\frac{\mathrm{icorr}}{T}=\mathrm{Log}\frac{\mathrm{R}}{Nh}+\frac{\Delta Sa}{2.303R}-\frac{\Delta Ha}{2.303RT}$$

where A is the pre-exponential factor, N is Avogadro’s number (6.022 × 1023 mol^−1^), h is the Planck’s constant (6.626176 × 10^−34^ Js), ΔS_a_ is the activation entropy, ΔH_a_ is the activation enthalpy, and Ea is the activation energy of the corrosion process. Figure 8 plots these equations as log i_corr_ and log i_corr_/T against the reciprocal of absolute temperature, respectively. The values of E_a_ and A were determined using the slope (− E_a_ /2.303R) and intercept (log A).

The values of ΔH and ΔS were found using the slope of (− ΔH_a_/2.303 R), and an intercept [(log (R/Nh) + (ΔS_a_/2.303 R)] of Eq. (9). Table 5 presents the results that were gathered. The protective approach is determined by the adsorption of inhibitors on the CS surface. The inhibitor may be applied chemically or physically to metal surfaces. E_a_ varies from 5 to 40 kJ mol^−1^ for physical adsorption and from 40 to 80 kJ mol^−1^ for chemical adsorption. Since E_a_, when there is CBA, it is greater than that in its absence. According to the findings, the examined CBA slows down the rate at which the CS dissolves. Consequently, CBA may be a potent inhibitor to stop CS corrosion in 1.0 M HCl. Therefore, the adsorption mode fell inside the physical one in 1.0 M HCl with an inhibitor (E_a_ = 33.65 kJ mol^−1^). The inhibitor efficiency test findings (Table 4) support this. The percentage η values were low and fell as the temperature rose. Positive ΔH_a_ values indicate an endothermic dissolving process in aqueous solutions, which may be caused by weak adsorption and inhibitor molecule desorption on the surface of the CS. The behavior of ΔH_a_ values was analogous to that of E_a_. The elevated ΔH_a_ values in the inhibited (192 µmol L^−1^ CBA) solution are produced by the high corrosion-inhibiting action of CBA. Additionally, Fig. 10 (“Adsorption isotherm” section) shows the relationship between log i_corr_/T against T^−1^/K^−1^, which is used to govern the values of ΔH* and ΔS*. Positive ΔH* values are discovered in the data, showing that the dissolution of the CS is endothermic. CS is corroding at a slower rate in 1.0 M HCl, as demonstrated by given that the value of ΔH* for CBA is larger compared to that of the blank solution^55^. Additionally, negative ΔS* values imply that the rate at which activated complexes are produced is determined by 1.0 M HCl association rather than only dissociation, which lessens disorder^55^.

**Table 5:** Kinetic characteristics in 1.0 M HCl with and without CBA.

Solution (µmol L^−1^)	$\Delta Ha$ (kJ mol^−1^)	$\Delta Sa$ (J mol^−1^ K^−1^)	E_a_ (kJ mol^−1^)
0.00	13.38	− 199.8	14.49
192	32.44	− 145.2	33.65

#### Electrochemical impedance spectroscopy (EIS)

**Fig. 9:**
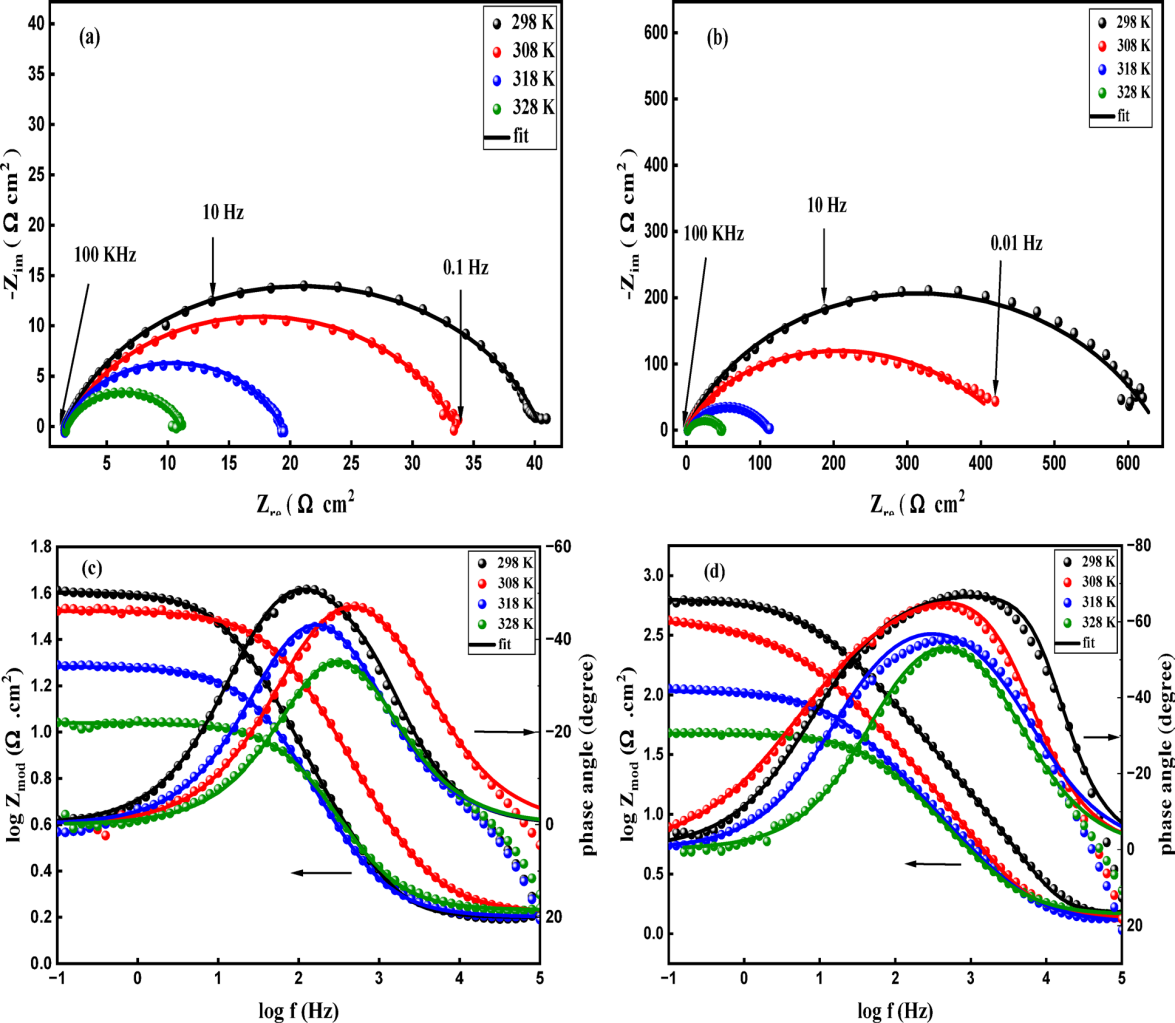
Nyquist plots (**a**,**b**), and (**c**,**d**) Bod plots of CS EIS data in a 1.0 M HCl solution obtained at various temperatures with and without the CBA, respectively.

At different temperatures (298, 308, 318, and 328 K), EIS tests are conducted in a 1.0 M HCl solution with and without CBA. The Nyquist plot of a single capacitive loop with a large polarization resistance is shown in Fig. 9a and b. The Nyquist graphic demonstrates how the semicircle diameter shrinks when CBA desorbs off the CS surface at high temperatures. Table 6 illustrates that the R_ct_ value rises when CBA is added to 1.0 M HCl at any temperature, in contrast to the CBA-free solution at the same temperature. The drop in R_ct_ values of CBA by increasing temperature specifies that the desorption of CBA from the CS surface causes worse surface coverage of CBA on CS, a greater rate of corrosion, and ultimately reduced inhibitory efficiency.

**Table 6:** EIS fitting parameters were found for a CS in a 1.0 M HCl solution at various temperatures, both with and without the CBA.

Temperature (K)	R_s_ (Ω cm^2^)	R_ct_ (Ω cm^2^)	CPE_dl_ (Ω^−1^ s^n^ cm^−2^)	n	$\Theta$	ղ_EIS_(%)
0.00 µmol L^−1^						
298	1.549	38.87	657.0 × 10^−6^	0.792	–	–
308	1.659	31.86	686.6 × 10^−6^	0.785	–	–
318	1.568	17.64	703.2 × 10^−6^	0.790	–	–
328	1.700	9.287	749.9 × 10^−6^	0.803	–	–
192 µmol L^−1^						
298	1.257	600.7	67.61 × 10^−6^	0.800	0.935	93.52
308	1.226	371.2	190.6 × 10^−6^	0.771	0.914	91.41
318	1.233	107.3	295.6 × 10^−6^	0.751	0.835	83.56
328	1.437	46.06	341.5 × 10^−6^	0.785	0.798	79.83

#### Adsorption isotherm

**Fig. 10:**
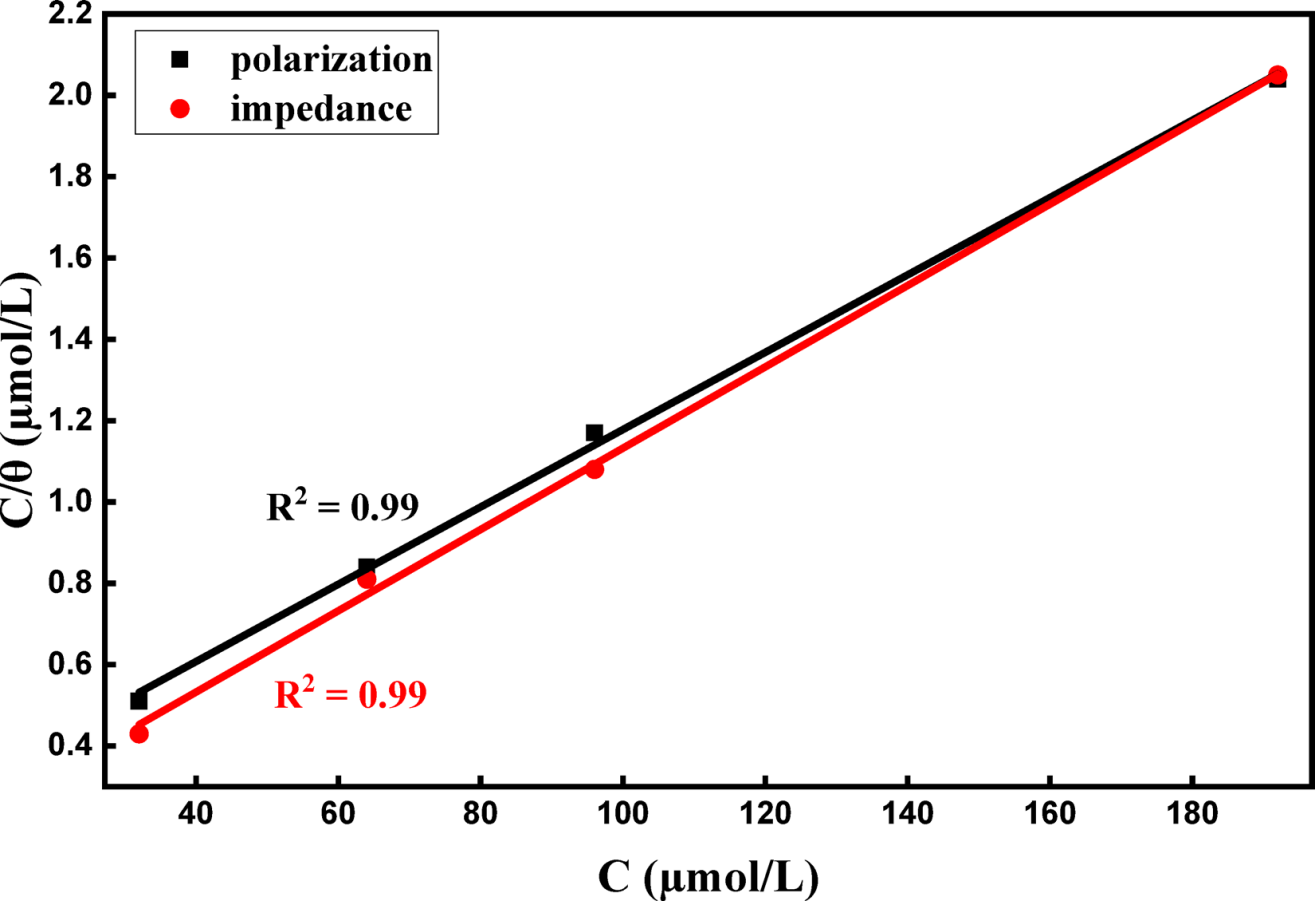
Langmuir adsorption isotherm to the impedance and polarization measurements for CS in a 1.0 M HCl at 298 K with different CBA concentrations.

The next equation illustrates how organic molecules (CBA) substitute water molecules and explains why CBA molecules adsorb at the CS/1.0 M HCl interface:

11 $${\mathrm{CBA}}_{{{\mathrm{sol}}}} + {\mathrm{nH}}_{2} {\mathrm{O}}_{{{\mathrm{ads}}}} \leftrightarrow {\mathrm{CBA}}_{{{\mathrm{ads}}}} + {\mathrm{nH}}_{2} {\mathrm{O}}_{{{\mathrm{sol}}}}$$

where n is the quantity of water molecules replaced, and CBA_sol_ and CBA_ads_ stand for the CBA compounds in the solution (liquid phase) and adsorbed on CBA-CS (adsorption phase), respectively^56^. The main aspect prompting an organic material’s capacity to lessen corrosion is its capacity to adsorb onto the CBA-CS surface. Understanding the method of adsorption was essential to understanding how the CBA molecules interacted with the alloy’s surface. Multiple isotherms of adsorption were applied to examine the analyzed CBA. The adsorption process of CBA at the CS/solution contact was quite similar to the Langmuir adsorption isotherm. Plotting data against C_inh_ using both polarization and EIS methods produces straight lines with R^2^ values of 0.99 (Fig. 10). The Langmuir adsorption isotherm is defined as^57^:

12 $${\mathrm{C}}_{{{\mathrm{inh}}}} /\uptheta = 1/{\mathrm{K}}_{{{\mathrm{ads}}}} + {\mathrm{C}}_{{{\mathrm{inh}}}}$$

where C_inh_ is the CBA concentration, K_ads_ is the adsorption equilibrium constant, and θ is the surface coverage (θ = (ղ (%) /100). The next equation was used to govern the typical adsorption-free energy, or ΔG°_ads,_ at 298 K^57^:

13 $$\Delta {\mathrm{G}}_{{{\mathrm{ads}}}}^{\circ } = - {\mathrm{RTLn}}\left( {55.5\,{\mathrm{K}}_{{{\mathrm{ads}}}} } \right)$$

wherever R is the universal gas constant, T is the Kelvin temperature, and the molar concentration of water is 55.5. It is well known that physisorption works well for ΔG°_ads_ with values less than − 20 kJ mol^−1^, which require chemisorption, whereas those with values larger than − 40 kJ mol^−1^ require it^58^. The ΔG°_ads_ value showed the type of adsorption. Based on the impedance and polarization measurements, the CBA’s computed ΔG°_ads_ value is − 31.30 kJ mol^−1^ and − 31.13 kJ mol^−1^, respectively. A negative ΔG°_ads_ value (Table 7) indicates that CBA adsorption occurs spontaneously on the CS surface, indicating that physisorption is the prime mechanism of CBA adsorption on the CS surface. Considering measurements of polarization and impedance, the equilibrium constant (K_ads_) can be computed and determined to be 105.15 and 100, respectively. The strength of the adsorption forces between the CBA molecule and the CS surface can be determined by means of the K_ads_ value. The larger K_ads_ value established the inhibitor’s increased adsorption ability on the CS surface.

**Table 7:** The equilibrium constant of the inhibitor (K_ads_) and the standard free energy of adsorption (ΔG°_ads_) for CS in 1.0 M HCl at 298 K.

Material	K_ads_ (L mol^−1^)		ΔG°_ads_ (kJ mol^−1^)	
	Polarization	Impedance	Polarization	Impedance
CS	105.15	100	− 31.30	− 31.13

#### Surface topography analysis

**Fig. 11:**
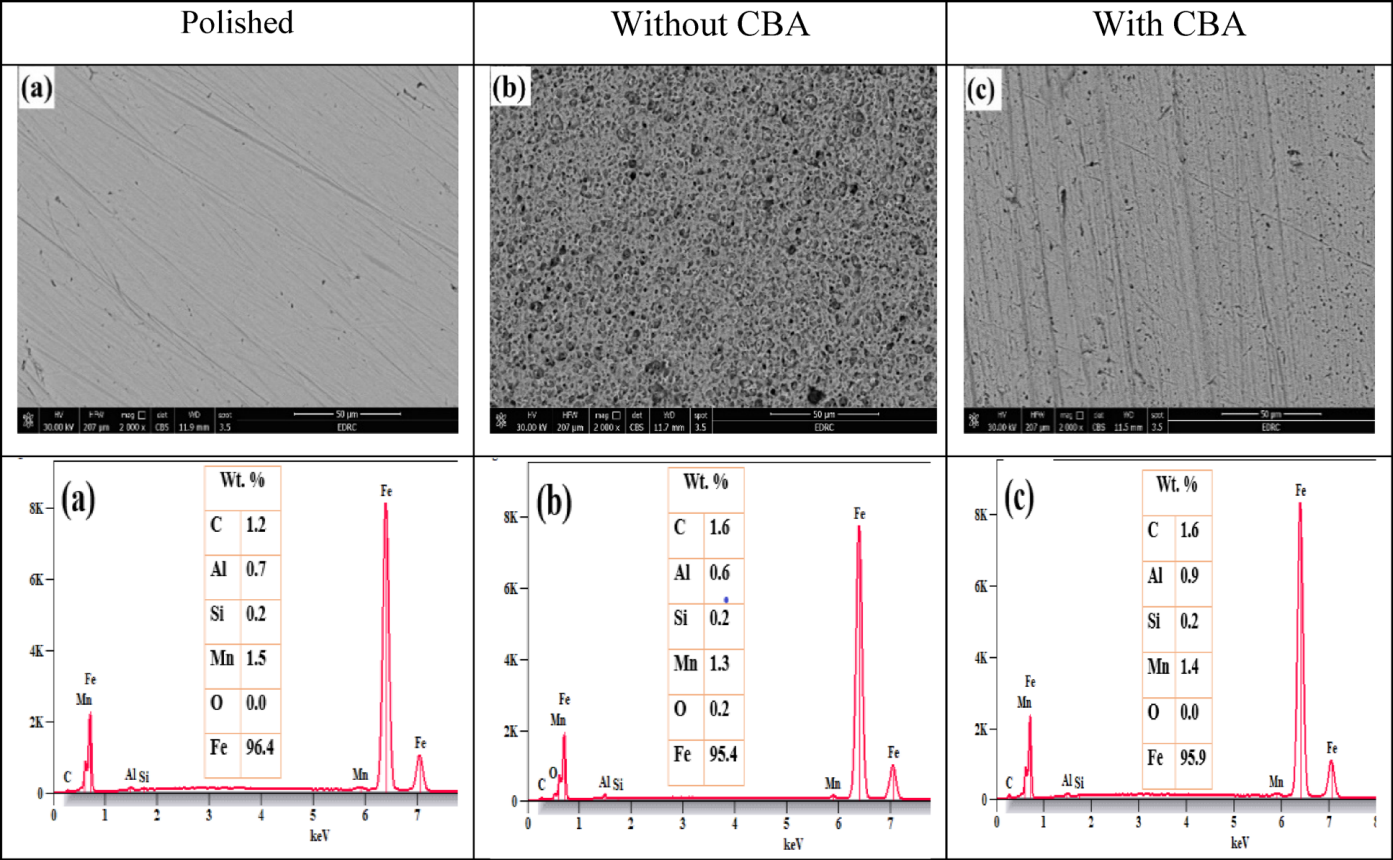
Surface morphology and EDX of CS (**a**,**a-**) polished, (**b**,**b-**) immersed in 1.0 M HCl without CBA, and (**c**,**c-**) immersed in 1.0 M HCl with CBA for 8 h.

**Fig. 12:**
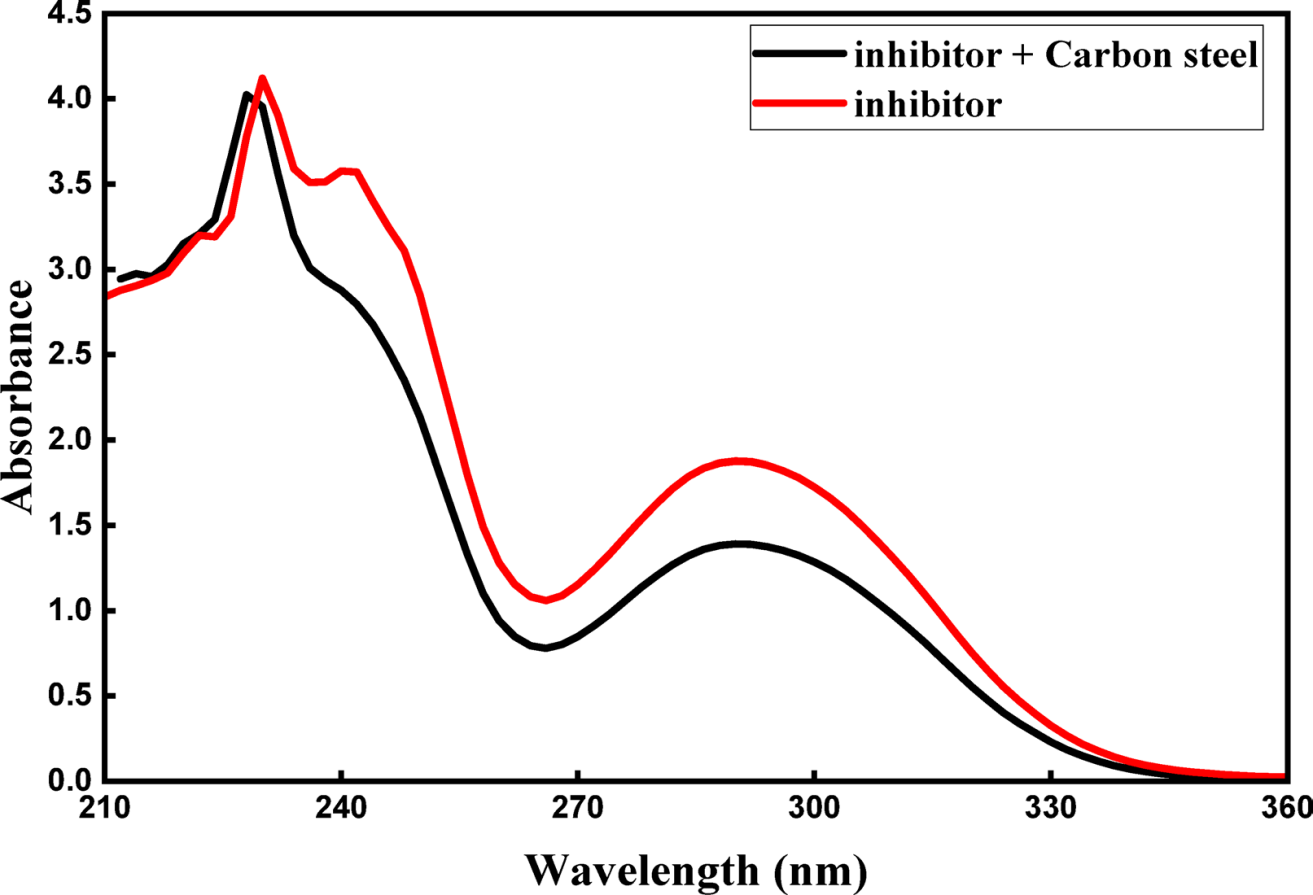
UV spectra of CBA prior to and following the 8-h CS immersion.

SEM comparison of the surface topography of CS samples treated with and without 192 µmol L^−1^ of CBA corrosion inhibitor after 8 h in 1.0 M HCl is shown in Fig. 11. After an 8-h immersion in 1.0 M HCl without the addition of CBA corrosion inhibitor, Fig. 11b illustrates a significant corrosion attack on the CS substrate. The corrosion produced on the CS surface was significantly reduced when the CBA corrosion inhibitor was added, as shown in Fig. 11c. To confirm the CBA inhibitor adsorption on the surface of CS, the UV spectra of CBA were examined before and after the corrosion study (Fig. 12). The corrosive environment in which the CS sample was not submerged had a higher absorbance than the condition in which the sample was submerged for a full eight hours, according to the UV spectra. After the CS sample was submerged, some of the molecules in the CBA solution were adsorbed on its surface. CBA has good corrosion inhibition efficiency, as evidenced by its successful adsorption on the CS sample.

**Fig. 13:**
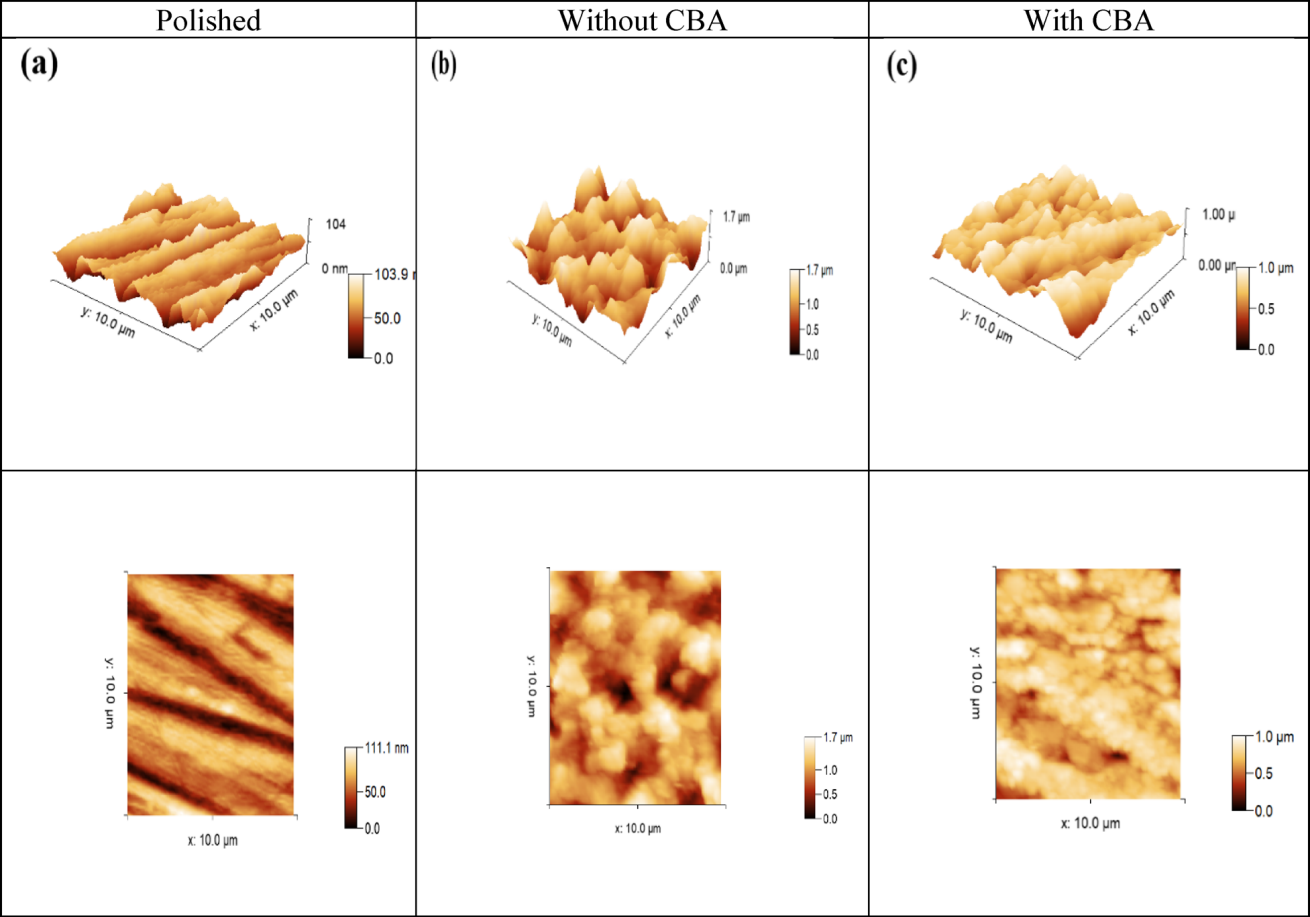
AFM pictures of CS: (**a**) Polished CS, (**b**) CS submerged in 1.0 M HCl; (**c**) CS submerged in 1.0 M HCl at 298 K with 192 µmol/L CBA.

The surface morphological evolution of CS caused by corrosion and inhibitor adsorption under various conditions was qualitatively investigated by AFM, as illustrated in Fig. 13. The absence of corrosion damage is indicated by the polished CS surface’s comparatively smooth and uniform shape (Fig. 13a), which includes shallow grooves from mechanical polishing. The CS surface deteriorates significantly after being submerged in 1.0 M HCl without CBA (Fig. 13b), exhibiting noticeable changes with a mean surface roughness of 232.4 nm, characterized by deep pits and uneven features. The aggressive attack of H^+^ and Cl^−^ ions, which accelerates iron dissolution and promotes localized corrosion, is responsible for this morphological deterioration. In contrast, the surface of CS submerged in 1.0 M HCl containing CBA (Fig. 13c) shows a considerably smoother and more uniform morphology with a discernible reduction in corrosion defects. Effective surface protection is suggested by the surface appearance, which closely mirrors that of the polished sample. However, upon exposure to the electrolyte containing 192 µmol L^−1^ of CBA on the steel surface, the mean surface roughness reduced to 100.9 nm (Fig. 12c). This improvement is owing to the adsorption of CBA molecules onto the *CS* surface, which forms a barrier that blocks active sites and keeps corrosive species from penetrating. Our AFM results strongly support the inhibitive performance of CBA seen in electrochemical investigations.

#### Contact angle (CA)

**Fig. 14:**
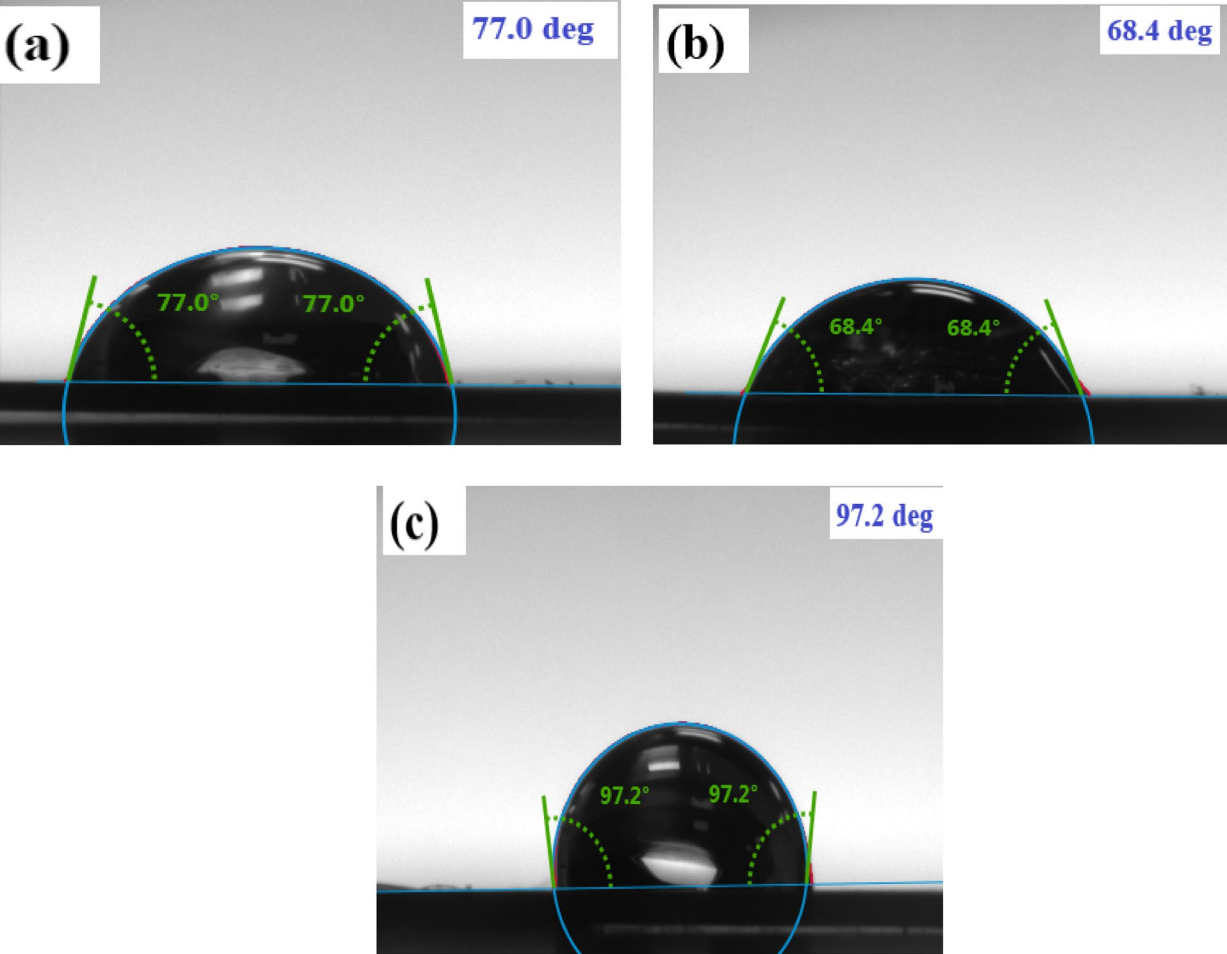
Contact angle measurements and pictures of (**a**) a polished CS surface; (**b**) after 6 h of immersion in 1.0 M HCl (blank); and (**c**) after 8 h of immersion in 1.0 M HCl with 192 µmol/L of CBA.

To verify that the CBA layer had created a protective shield on the CS, approach contact angle tests were performed. A 4.0 μL drop of water was placed on the CS surface to get this measure. The CA evaluations for the refined CS sample (Fig. 14a) and the CS submerged in HCl solution with and without CBA inhibitor (Fig. 14b,c) are examined. After the polished CS sample (Fig. 14a) was immersed in 1.0 M HCl, its CA declined from 77.0° to 68.4° (Fig. 14b). This decline shows that the 1.0 HCl corrosive action has enhanced the surface’s wettability. But the contact angle of the metal dipped in the CBA rose to 97.2° (Fig. 14c). The adsorption of CBA on the CS surface is shown by this notable increase in CA, which shows a decrease in wettability and an increase in hydrophobicity. The creation of a protective layer on the CS sample efficiently decreases the surface’s interaction with water by serving as a barrier and shielding the 1.0 M HCl film from being harmed by the hazardous environment, as seen by the increase in CA with the CBA overview. This protective layer illustrates how well the CBA inhibitor prevents degradation.

#### DFT studies

**Fig. 15:**
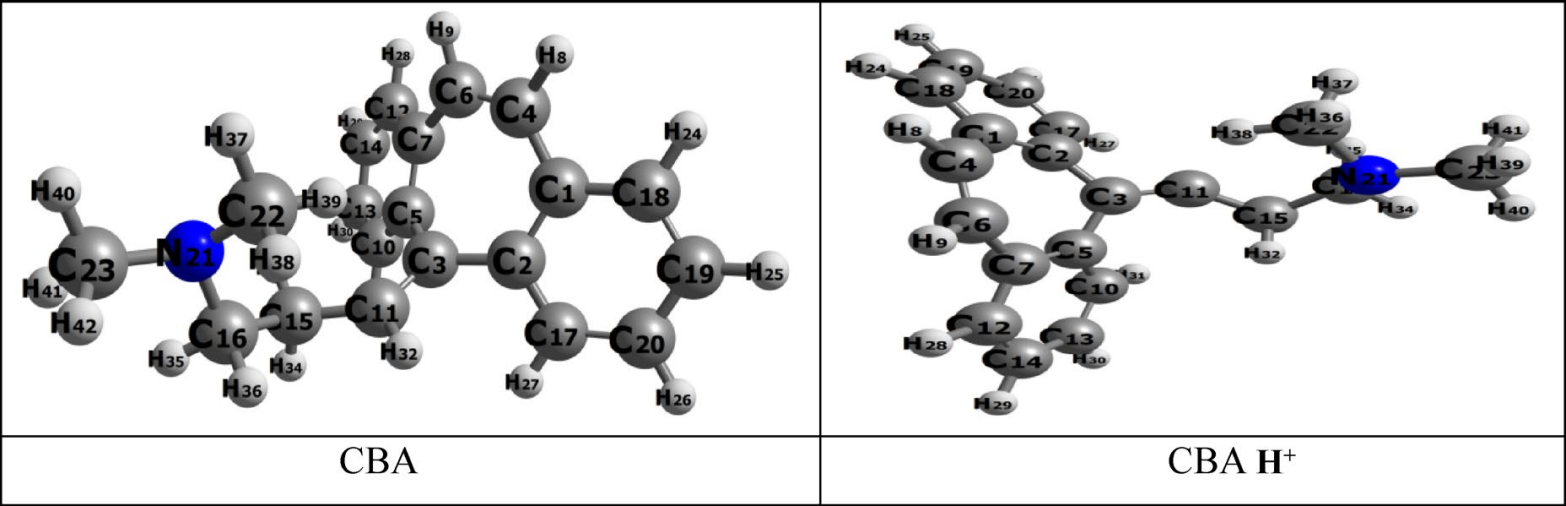
Optimized structure of the CBA and CBA** H**^**+**^ using the B3LYP/6-311++ G(d,p) basis set.

**Fig. 16:**
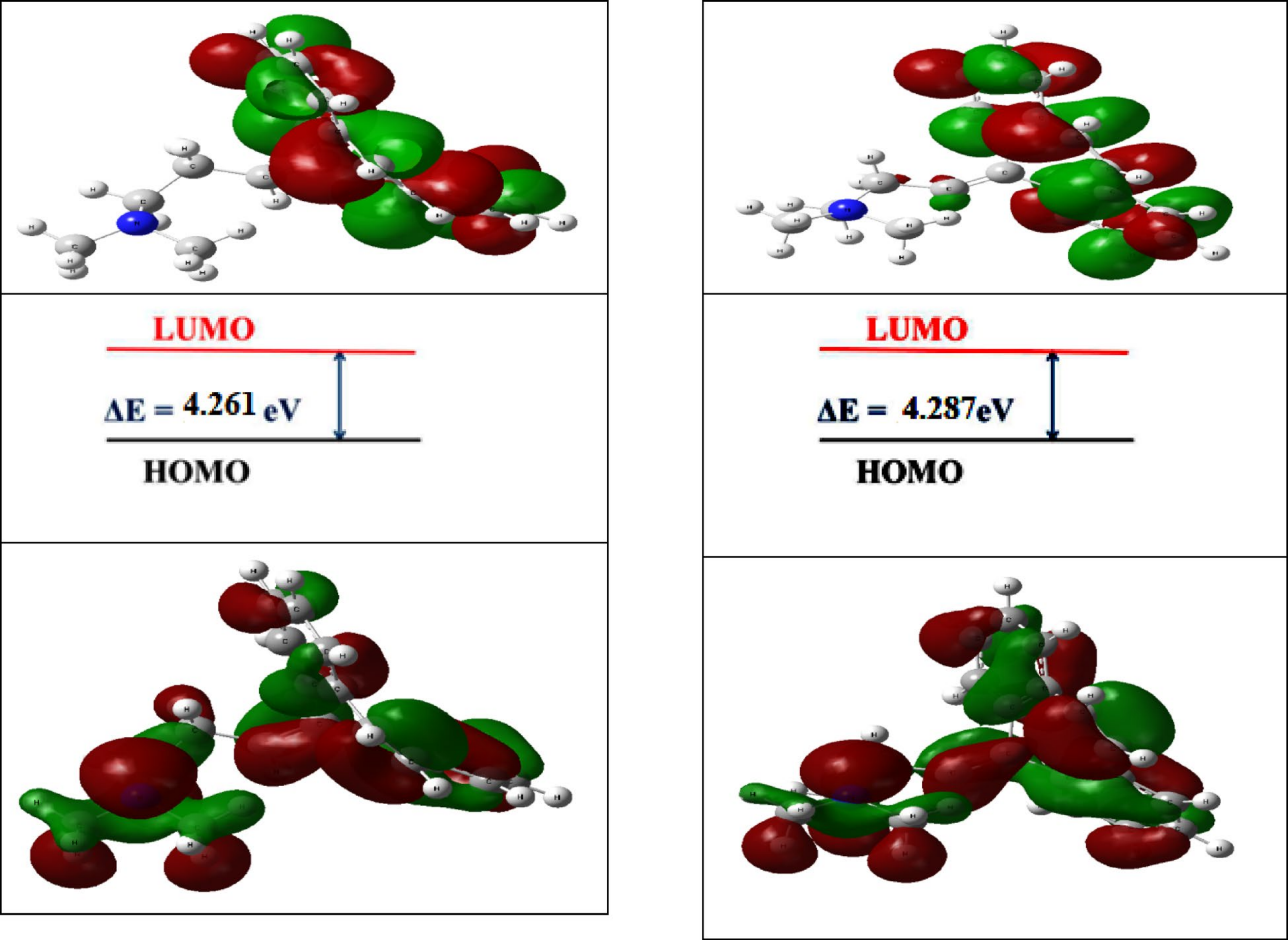
Using the B3LYP/6-311++ G(d,p) basis set, the frontier molecular orbitals HOMO and LUMO of the CBA and CBA H^+^.

**Fig. 17:**
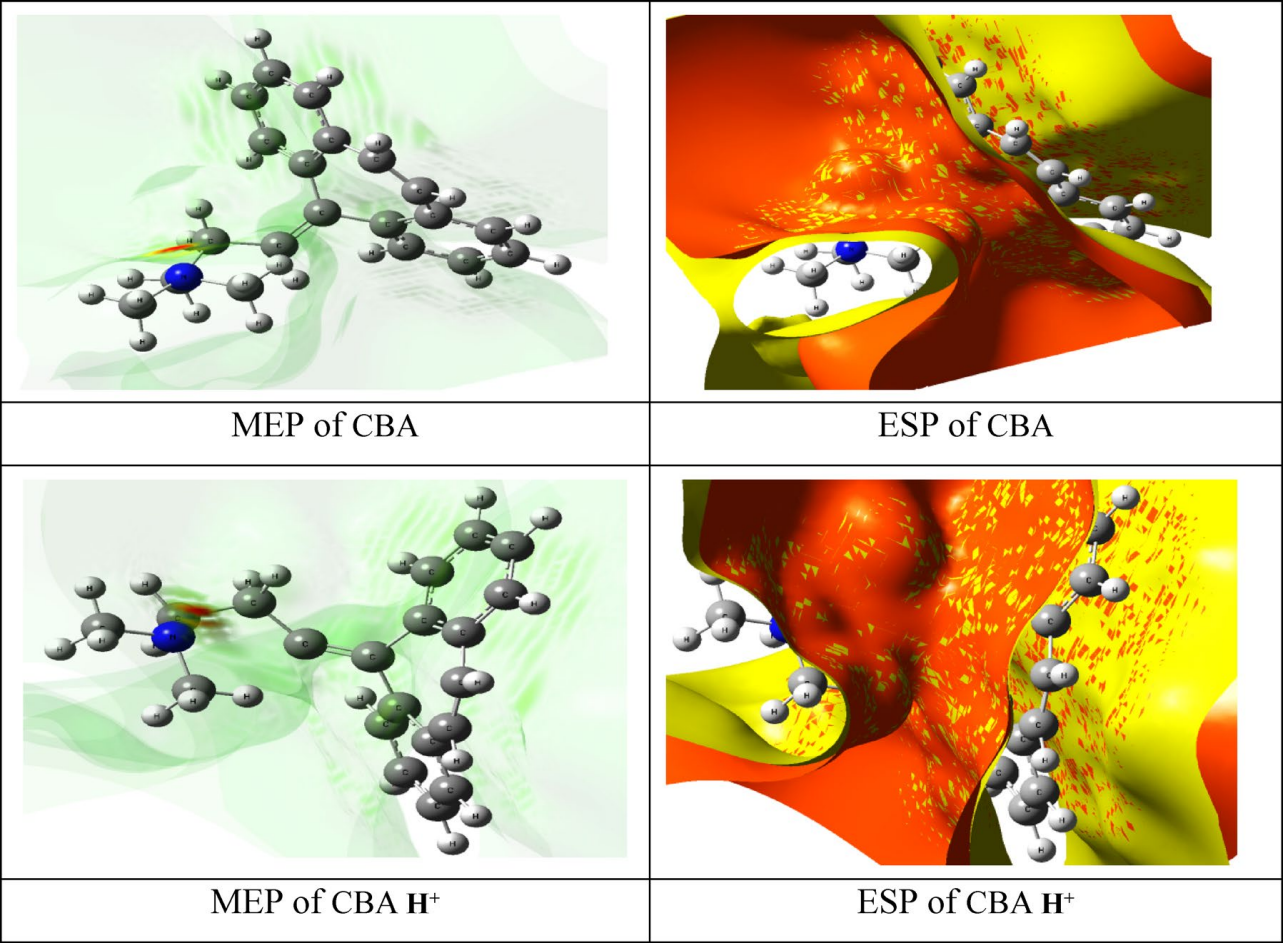
The ESP and MEP distribution of the neutral CBA and CBA H^+^ using the basis set B3LYP/6-311++ G(d, p).

The electronic reactivity and inhibitory performance of CBA were examined using Density Functional Theory (DFT) computations. Table 8 summarizes the computed quantum chemical descriptors, and Figs. 15 and 16 display the optimized structures and frontier molecular orbitals (HOMO and LUMO) of neutral and protonated CBA (CBA and CBAH^+^). The HOMO density is mainly localized on aromatic rings and heteroatoms, identifying these regions as preferential electron-donating sites toward the carbon steel surface, consistent with the experimental inhibition results^59–62^. Improved molecular reactivity and inhibition efficiency are indicated by a smaller HOMO–LUMO energy gap (ΔE). Additional descriptors influencing inhibitor–metal interactions were derived using E_HOMO_ and E_LUMO_, which are related to ionization potential and electron affinity^63,64^.The relatively low dipole moment (μ), low electronegativity (χ), high softness (S), and low hardness (η) confirm the strong electron-donating ability of CBA, with the protonated form (CBAH^+^) exhibiting higher softness and stronger surface interaction (Table 8)^65,66^. The electrophilicity (ω) and nucleophilicity (ε) indices indicate predominantly nucleophilic behavior, while high electron-donating power (ω^−^), negative back-donation energy (ΔE_back < 0), and acceptable electron transfer fraction values (ΔN < 3.6) further support electron donation from CBA to Fe atoms and stable adsorption. Local reactivity was evaluated using Fukui functions (Eqs. 14–16)^28,29^, with the results summarized in Table 9.

14 $$f_{k}^{ + } = q_{k} \left( {{\mathrm{N}} + 1} \right) - q_{k} \left( {\mathrm{N}} \right)\quad \quad \left( {\text{nucleophilic attack}} \right)$$

15 $$f_{k}^{ - } = q_{k} \left( {\mathrm{N}} \right) - q_{k} \left( {{\mathrm{N}} - 1} \right)\quad \quad \left( {\text{electrophilic attack}} \right)$$

16 $$\Delta f_{k} = f_{k}^{ + } - f_{k}^{ - }$$

where the charge values of atoms *k* for neutral, anionic, and cationic states are denoted by *q*_*k*_(N), *q*_*k*_(N + 1), and *q*_*k*_(N − 1). Morell et al.’s dual descriptor, Δ*f*_*k*_, is computed as the difference between the nucleophilic and electrophilic Fukui functions^67^. For neutral CBA, N21, C16, and C23 were identified as nucleophilic centers, whereas C7 and C8 were more susceptible to electrophilic attack; protonation alters the reactive pattern, with C19 and C3 acting as nucleophilic sites and C16 and N21 as electrophilic centers in CBAH^+^. The electrostatic potential (ESP) and molecular electrostatic potential (MEP) maps (Fig. 17) show high electron density localized on heteroatoms, particularly nitrogen, confirming their role as the primary adsorption sites and supporting the proposed inhibition mechanism^68,69^.

**Table 8:** Computed quantum chemical descriptors for the studied neutral CBA and protonated.

Descriptors	Equations	CBA	CBA H^+^
Energy of highest occupied molecular orbital (E_HOMO_), (eV)		− 5.777	− 5.806
Energy of lowest unoccupied molecular orbital (E_LUMO_), (eV)		− 1.516	− 1.519
Energy gap ΔE	(LUMO–HOMO)	4.261	4.287
Dipole moment, (µ), (Debye)		0.533	0.844
Ionization energy (I) (ev)	$I={-E}_{HOMO}$	5.777	5.806
Electron affinity ($Y$) (ev)	$Y={-E}_{LUMO}$	1.516	1.519
Electronegativity (*ϕ*)	*ϕ* = $\frac{I+Y}{2}$	3.646	3.662
Global hardness *ψ*	*ψ* = $\frac{I-Y}{2}$	2.131	2.143
Global softness (S)	s = $\frac{1}{\psi }$	0.469	0.467
Global electrophilicity (ω)	ω = ϕ^2^/2*ψ*	3.120	3.128
Global nucleophilicity (ε)	$\upvarepsilon =\frac{1}{\upomega }$	0.321	0.320
Electroaccepting (ω^+^) power	${\omega }^{+}=\frac{{\left(I+3A\right)}^{2}}{16\left(I-A\right)}$	1.564	1.566
Electrodonating (ω^−^) power	${\omega }^{-}=\frac{{\left(A+3I\right)}^{2}}{16\left(I-A\right)}$	5.210	5.229
Net electrophilicity (Δω ± = ω^+^ + ω^−^)	(Δω^±^ = ω^+^ + ω ^−^)	6.774	6.795
Fraction of transferred electrons (ΔN)	$\Delta N=\frac{{\phi}_{\mathrm{F}\mathrm{e}-}{\phi}_{\mathrm{i}\mathrm{n}\mathrm{h}}}{2({\psi}_{\mathrm{F}\mathrm{e}}+{\psi}_{\mathrm{i}\mathrm{n}\mathrm{h}})}$	0.1397	0.1364
Back-donation energy ΔE back-donation (ev)	${\Delta \mathrm{E}}_{\mathrm{b}\mathrm{a}\mathrm{c}\mathrm{k}-\mathrm{d}\mathrm{o}\mathrm{n}\mathrm{a}\mathrm{t}\mathrm{i}\mathrm{o}\mathrm{n} }=- \frac{\psi }{4}$	− 0.533	− 0.536
Metal/inhibitor interaction energy ΔE_Metal/inhibitor_ (ev)	${\Delta {\rm E}}_{steel/\mathrm{C}\mathrm{B}\mathrm{A}}= \frac{{({\chi}_{Fe}-{\chi}_{inh)}}^{2}}{4({\eta}_{Fe}+{\eta}_{inh})}$	0.0573	0.0548

CBA H^+^ inhibitor in the gas phase.

**Table 9:** Fukui indices of the studied CBA and CBA H^+^

CBA				CBA H^+^			
Atoms	${f}_{k}^{+}$	${f}_{k}^{-}$	$\Delta {f}_{k}$	atoms	${f}_{k}^{+}$	${f}_{k}^{-}$	$\Delta {f}_{k}$
N (21)	0.001	0.061	− 0.06	N (21)	0.01	0.005	0.005
C (18)	0.029	− 0.003	0.032	C (18)	0.009	0.006	0.003
C (19)	0.028	− 0.002	0.03	C (19)	0.012	0.005	0.007
C (11)	0.027	0.001	0.026	C (11)	0.003	0.01	− 0.007
C (13)	0.023	0.002	0.021	C (13)	0.006	0.007	− 0.001
C (16)	0.037	0.004	0.033	C (16)	0.022	− 0.001	0.023
C (17)	0.011	− 0.006	0.017	C (17)	0.005	0.016	− 0.011
C (4)	0.014	− 0.01	0.024	C (4)	0.005	0.007	− 0.002
C (7)	0.008	0.007	0.001	C (7)	0.006	0.006	0
C (1)	0.014	0	0.014	C (1)	0.004	0.011	− 0.007
C (2)	0.009	0.01	− 0.001	C (2)	0.005	0.01	− 0.005
C (3)	0.019	0.009	0.01	C (3)	0.013	0.001	0.012
C (6)	0.01	− 0.001	0.011	C (6)	− 0.003	0.023	− 0.026
C (7)	0.019	0.001	0.018	C (7)	0.004	0.014	− 0.01
C (10)	0.022	0.002	0.02	C (10)	0.005	0.012	− 0.007
C (5)	0.019	0	0.019	C (5)	0.003	0.013	− 0.01
C (20)	0.022	0.003	0.019	C (20)	0.003	0.013	− 0.01
C (23)	0.037	0.005	0.032	C (23)	0.012	0.027	− 0.015
C (22)	0.003	− 0.003	0.006	C (22)	− 0.004	0.029	− 0.033
C (12)	0.006	0.004	0.002	C (12)	0.004	0.014	− 0.01
C (14)	0.01	− 0.001	0.011	C (14)	0.003	0.016	− 0.013
C (15)	0.009	0.008	0.001	C (15)	0.002	0.015	− 0.013

The molecular electrostatic potential (MEP) and electrostatic potential (ESP) maps (Fig. 17) show high electron density localized on heteroatoms, particularly nitrogen, confirming their role as the primary adsorption sites and supporting the proposed inhibition mechanism^68,69^.

#### Natural bond orbital (NBO) analysis

Charge transfer and electronic stabilization in CBA were evaluated using Natural Bond Orbital (NBO) analysis. Electron delocalization from filled bonding (BD) and lone pair (LP) orbitals to empty antibonding (BD*) or Rydberg (RY*) orbitals was quantified using second-order perturbation energies (E^2^), which shed light on hyperconjugation, resonance, and non-covalent interactions controlling molecular stability^70–73^. The biggest contributions to stabilization (Table 10) come from LP(1) N21 → π* N21–C16 (46.74 kcal mol^−1^) and several π → π* interactions, including π C10–C11 → π* C4–C15 (55.13 kcal mol^−1^), π C6–C12 → π* C6–C20 (64.52 kcal mol^−1^), and π C10–C24 → π* C18–C24 (60.05 kcal mol^−1^). These interactions emphasize the dominant role of nitrogen-centered electron delocalization and π-electron conjugation in stabilizing the CBA molecule. Overall, the high E^2^ values indicate strong electronic stabilization, which correlates well with the experimentally observed inhibition efficiency and supports the proposed adsorption mechanism inferred from AFM, EIS, and surface analyses.

**Table 10:** Fock matrix in NBO basis of CBA molecule at B3LYP/6-311++ G (d, p) level in the gas phase analyzed using second-order perturbation theory.

Compound	Donor	Acceptor	E^(2)a^ (kcal/mol)	Occupancy
CBA	πC3–C13	π*C15–C11	33.52	1.88
	πC4–C15	π*C16–C12	35.13	1.75
	πC5–C14	π*C17–C20	43.52	1.67
	πC10–C11	π*C4–C15	55.13	1.76
	πC6–C12	π*C7–C20	64.52	1.68
	πC1–C20	π*C2–C18	46.13	1.75
	πC22–C23	π*C17–C13	55.05	1.65
	LP (1) N21	π*C16–N21	46.74	1.87
	πC22–N21	π*C22–N21	26.58	1.82
	πC23–N21	π*C14–C6	16.47	1.94
	πC14–C20	π*C23–C15	31.04	1.84
	πC15–C19	π*C13–C26	49.83	1.75
	π*C23–C15	π*C22–C17	18.81	1.88
	π*C10–C24	π*C18–C24	60.05	1.70

### Limitations of the study

The observed inhibitory performance is restricted to the examined concentration and temperature ranges because this investigation was conducted utilizing short-term electrochemical and gravimetric experiments in 1.0 M HCl under controlled laboratory settings. Environmental and economic factors, behavior in dynamic or industrial settings, and long-term stability were not evaluated. Before these findings can be applied in real-world settings, more research is necessary.

### Inhibition mechanism

A series of connected processes that take place at the metal/electrolyte interface can be used to explain the cyclobenzaprine (CBA) corrosion inhibition mechanism on CS in 1.0 M HCl. Carbon steel first experiences cathodic hydrogen evolution (2H^+^ + 2e^−^ → H_2_) and anodic dissolution (Fe → Fe^2+^ + 2e^−^) in the acidic media. Initial physical adsorption results from the protonation of inhibitor molecules in the acidic solution upon the addition of CBA. These molecules are then electrostatically drawn to the negatively charged sites on the steel surface.

CBA molecules therefore exhibit stronger adsorption through π–metal interactions involving the aromatic rings and donor–acceptor interactions between the lone pair electrons of the nitrogen atom and the unoccupied *d*-orbitals of iron atoms. A dense and adherent protective film gradually forms on the steel surface as a result of this combination of physical and chemical adsorption.

The adsorbed CBA layer functions as a barrier that limits the flow of aggressive chloride ions toward the metal surface, suppresses charge transfer processes, and prevents active corrosion sites as surface coverage increases. Electrochemical impedance measurements show a drop in double-layer capacitance and an increase in charge transfer resistance.

In line with the mixed-type inhibition characteristic of CBA, the formed inhibitor film stabilizes the metal surface in the final stage while concurrently delaying anodic metal dissolution and cathodic hydrogen evolution events. Strong adsorption and advantageous molecular orientation of CBA on the Fe (111) surface are confirmed by surface morphology studies and computational results, which further validate the suggested process.

## Conclusion

- Using complementary experimental and computational methods, cyclobenzaprine hydrochloride (CBA) was successfully assessed as a corrosion inhibitor for CS in 1.0 M HCl solution.
- CBA significantly lowers the rate of CS corrosion, with inhibition efficiencies exceeding 95% at the optimal concentration, according to weight loss and electrochemical measurements.
- Potentiodynamic polarization results showed that CBA functions as a mixed-type inhibitor, preventing anodic metal dissolution and cathodic hydrogen evolution processes without altering the underlying corrosion mechanism.
- The formation of a compact and protective adsorbed layer was confirmed by electrochemical impedance spectroscopy, which showed a notable reduction in double-layer capacitance and an increase in charge transfer resistance when CBA was present.
- The inhibitor follows the Langmuir adsorption isotherm, as indicated by the adsorption studies.
- Negative free energy of adsorption values suggest that spontaneous adsorption is dominated by physisorption, with partial chemisorption contributions.
- The adsorption mechanism was further supported by the reduction in inhibition efficiency with increasing temperature.
- In comparison to uninhibited steel, surface morphology and wettability analyses (SEM, AFM, contact angle, and UV–Vis spectroscopy) directly demonstrated effective surface coverage by CBA, leading to smoother surfaces and increased hydrophobicity.
- According to quantum chemical calculations, CBA’s favorable electronic properties, such as its high electron-donating capability, an appropriate HOMO–LUMO energy gap, and active adsorption centers on nitrogen atoms and aromatic π-systems, are linked to its high inhibition efficiency.
- NBO analysis verified strong intramolecular charge transfer and electronic stabilization.
- Overall, the findings show that, under carefully monitored laboratory settings, cyclobenzaprine hydrochloride effectively inhibits carbon steel corrosion in acidic solutions.
- Before applying these results to industrial systems, further research addressing long-term performance, environmental impact, and practical applicability is necessary, even if the findings point to the potential of medicinal compounds as corrosion inhibitors.

## Data Availability

All data generated or analyzed during this study are included in this published article.
